# Building on Surface-Active Ionic Liquids for the Rescuing of the Antimalarial Drug Chloroquine

**DOI:** 10.3390/ijms21155334

**Published:** 2020-07-27

**Authors:** Ana Teresa Silva, Lis Lobo, Isabel S. Oliveira, Joana Gomes, Cátia Teixeira, Fátima Nogueira, Eduardo F. Marques, Ricardo Ferraz, Paula Gomes

**Affiliations:** 1 LAQV-REQUIMTE, Departamento de Química e Bioquímica, Faculdade de Ciências, Universidade do Porto, P-4169-007 Porto, Portugal; up201303026@gmail.com (A.T.S.); up201606401@fc.up.pt (J.G.); catia.teixeira@fc.up.pt (C.T.); ricardoferraz@eu.ipp.pt (R.F.); 2 Global Health and Tropical Medicine, Instituto de Higiene e Medicina Tropical, Universidade Nova de Lisboa, P-1349-008 Lisboa, Portugal; lis.lobo@ihmt.unl.pt (L.L.); FNogueira@ihmt.unl.pt (F.N.); 3 CIQ-UP, Departamento de Química e Bioquímica, Faculdade de Ciências, Universidade do Porto, P-4169-007 Porto, Portugal; isabelmscoliveira@gmail.com (I.S.O.); efmarque@fc.up.pt (E.F.M.); 4 Ciências Químicas e das Biomoléculas, Escola Superior de Saúde, Politécnico do Porto, P-4200-072 Porto, Portugal

**Keywords:** antimalarial, chloroquine, ionic liquid, malaria, rescuing, repurposing, surface active

## Abstract

Ionic liquids derived from classical antimalarials are emerging as a new approach towards the cost-effective rescuing of those drugs. Herein, we disclose novel surface-active ionic liquids derived from chloroquine and natural fatty acids whose antimalarial activity in vitro was found to be superior to that of the parent drug. The most potent ionic liquid was the laurate salt of chloroquine, which presented IC_50_ values of 4 and 110 nM against a chloroquine-sensitive and a chloroquine-resistant strain of *Plasmodium falciparum*, respectively, corresponding to an 11- and 6-fold increase in potency as compared to the reference chloroquine bisphosphate salt against the same strains. This unprecedented report opens new perspectives in both the fields of malaria chemotherapy and of surface-active ionic liquids derived from active pharmaceutical ingredients.

## 1. Introduction

**Scheme 1:**
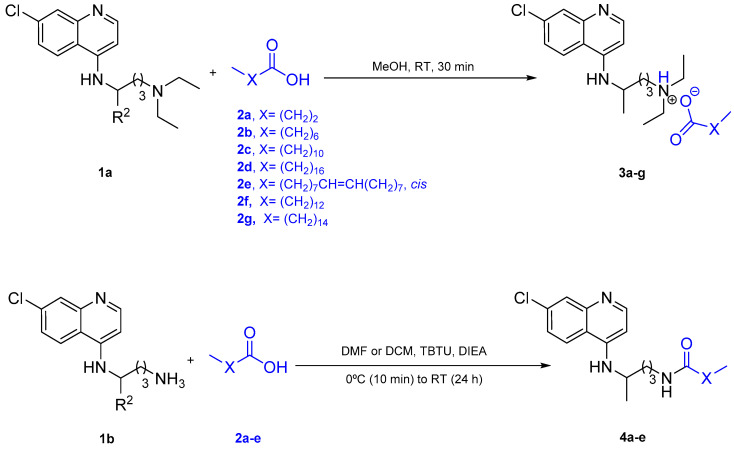
Routes towards organic salts **3a**–**e**, derived from chloroquine (CQ) (**1a**) and fatty acids **2a**–**e**, and their amide covalent counterparts **4a**–**e**, derived from CQ analogue **1b** and **2a**–**e**: (i) **1a** (1 molar equivalent, eq), **2a**–**e** (1 eq), methanol (MeOH), room temperature (RT), 30 min; (ii) **2a**–**e** (1 eq), *O*-(benzotriazol-1-yl)-*N,N,N’,N’*-tetramethyluronium tetrafluoroborate (TBTU; 1 eq), *N*-ethyl-*N,N*-diisopropylamine (DIEA, 2 eq), *N,N*-dimethylformamide (DMF), 0 °C, 10 min, then addition of **1b** (1 eq), RT, 24 h.

Ionic liquids (ILs) are gaining prominence as chemical entities of interest in medicinal chemistry, as well as pharmaceutical science and technology [1,2,3,4]. In fact, ILs can contribute to overcoming the undesirable features of conventional saline forms of active pharmaceutical ingredients (APIs), such as polymorphism, low solubility, and limited bioavailability [5,6]. As such, the development of ILs derived from APIs (API-ILs) is an appealing strategy towards the rescuing of drugs that are falling into disuse due to these and other detrimental traits. With this idea in mind, and following our previous promising findings on cinnamic acid conjugates of classical antimalarial drugs [7,8,9,10], we applied the API-IL concept onto such drugs, by disclosing room temperature ionic liquids (RTILs) derived from primaquine (PQ) and cinnamic acids as triple-stage antimalarial hits [11]. The most remarkable property of these RTILs was their increased activity against blood-stage malaria parasites, on which PQ has a practically negligible action [12]. This might be due to a more efficient interaction of RTILs with membranes of infected erythrocytes than with healthy ones, according to subsequent studies using model lipid membranes [13]. In view of this, we hypothesized that the combination of a blood schizonticide like chloroquine (CQ) with amphiphilic natural fatty acids might deliver new organic salts (Scheme 1) in the form of RTILs with enhanced blood-stage activity, possibly suitable for oral administration, as recently reported for lipid-based formulations of the lumefantrine docusate IL [14].

## 2. Results and Discussion

### 2.1. Chemical Synthesis and Thermal Stability

Commercially available CQ phosphate salt was first converted into the free amine form **1a**, which then reacted with fatty acids **2a**–**e** via an acid-base neutralization previously reported by us [11], to afford **3a**–**e** (Scheme 1). In parallel, **1b**, the primary amine analogue of **1a**, was prepared as previously reported by us [7] and conjugated with **2a**–**e** to produce amides **4a**–**e** for comparative assessment of in vitro antimalarial activity alongside salts **3a**–**e** (Scheme 1). The organic salts **3a**–**e** were obtained in nearly quantitative yields as colorless to pale yellow viscous liquids (i.e., **3a**–**e** are all RTILs), whereas amides **4a**–**e** were obtained as white to brownish-yellow solids in moderate to good yields (Table 1). Spectroscopic data, supplied as Supplementary Materials (SM), were in agreement with the expected structures. Notably, although proton nuclear magnetic resonance (^1^H-NMR) data are not conclusive regarding protonation of the basic groups in CQ (as spectra were acquired from hexadeuterated dimethylsulfoxide, DMSO-d6, which is an H-bond acceptor), it was possible to confirm complete transfer of the carboxylic acid proton to the aminoquinoline. This is because this proton was observed in the ^1^H-NMR spectra of solutions of the fatty acids **2a**–**e** in DMSO-d6, but not in the solutions of their respective CQ salts **3a**–**e** in the same solvent (Figure S1 of the SM). This is a remarkable finding, as it has been established by Stoimenovski et al. that a Δp*K*_a_ >10 is required to assure complete proton transfer between the proton donor and a tertiary amine [15].

All compounds were analyzed by simultaneous thermogravimetric analysis (STA), as given in detail in the SM, to assess their thermal stability, an important issue for APIs typically employed in the treatment of diseases, such as malaria, that are endemic to tropical and sub-tropical countries. The temperatures at which thermal degradation events were observed by STA are provided in Table 1, and the corresponding thermograms are provided in the SM. Data show that all the RTILs **3a**–**e** are slightly less thermally stable than the commercial CQ phosphate salt, but still remain unaltered up to about 90 °C (**3a**) or higher temperatures (**3b**–**e**). As expected, covalent amide analogues **4a**–**e** displayed higher thermal stability, with only one thermal degradation event occurring at temperature values that generally increased with the size of the alcanoyl chain, in the 291.3–336.5 °C range. Interestingly, RTIL **3a**–**c** presented two thermal degradation events, whereas **3d** and **3e** showed only one degradation event in the temperature range of the study (50–500 °C). The possibility that two degradation events might be occurring equally in these two cases, but at very close temperature values, cannot be ruled out at this stage. Still, data from STA clearly show that **3a**–**e** do not behave as the mere sum of their parent compounds, otherwise thermal degradation events would have been observed at the same values recorded for CQ and the relevant fatty acids **2a**–**e**. A deeper study to establish the mechanisms of thermal degradation of RTILs **3a**–**e** is under way, to enable the full profiling of these novel compounds.

### 2.2. Antimalarial Activity In Vitro

Compounds **3a**–**e** and **4a**–**e** were evaluated in vitro according to previously reported methods (cf. SM) [11], against a CQ-sensitive (3D7) and a CQ-resistant (Dd2) strain of *Plasmodium falciparum* (*Pf*). Interestingly, while all RTILs **3a**–**e** did not pose any significant solubility issues in the course of the in vitro assays, only amides **4a**–**c** were sufficiently soluble in the same conditions. Moreover, although accurate values for solubility (*log S*) are yet to be determined, RTILs **3a**–**e** were also slightly more soluble in water (albeit low) than their covalent analogues **4a**–**e**. More importantly, all RTILs displayed stronger activity than CQ, which is classically formulated as a phosphate salt, against both CQ-sensitive and CQ–resistant strains of *Pf*, the species responsible for the deadliest form of human malaria. The activities of **3a**–**e** were in the low- to mid-nanomolar ranges against the 3D7 and Dd2 strains, respectively, with **3c** (derived from dodecanoic, or lauric, acid **2c**) being the most potent of the set. This RTIL was nearly 20-fold more potent than its amide counterpart, **4c**, and over 10-fold more potent than the reference drug, against the 3D7 strain. Regarding activity against the Dd2 strain, **3c** was virtually comparable to **4c**, but nearly five-fold better than CQ phosphate. The in vitro activity of **3c** was further compared to those of the parent fatty acid, **2c**, and of an equimolar mixture of this acid with the commercial CQ phosphate. Although **2c** was completely devoid of antiplasmodial activity, its equimolar mixture with the reference drug was significantly less active than **3c** against both strains. These observations further reinforce that **3a**–**e** are chemical entities on their own, and not the simple mixtures of their parent compounds. These data also support that, as originally hypothesized, acid-base pairing of CQ with fatty acids delivers RTILs showing a clear gain over the parent drug concerning in vitro antimalarial activity.

One interesting observation from in vitro data above was that the laurate salt of CQ, **3c**, seemed to possess the optimal size for antiplasmodial activity, which is slightly decreased for compounds **3** with shorter and longer fatty acid chains. Considering the amphiphilicity of fatty acids and derivatives [16], and the probable enhancement of this property when pairing the fatty carboxylates with an amphiphilic cation-like protonated CQ, we anticipated that RTILs **3** could act as surface-active ionic liquids (SAILs) [17], and that such activity might be one factor contributing to their enhanced antiplasmodial potency as compared to the standard formulation of CQ. To test this hypothesis, we carried out surface tension studies as follows (cf. experimental details in the SM, Section 3).

### 2.3. Surface Tension Studies

The best antimalarial RTIL, **3c**, along with one shorter (**3b**) and two larger (**3f** and **3g**, respectively; X=(CH_2_)_12_ and X=(CH_2_)_14_ in Scheme 1) analogues, were further studied regarding their surface activity properties, to obtain a finer scrutiny of the effect of alkyl chain size variation on surface activity. The two additional compounds, **3f**,**g**, were synthesized as above described for **3a**–**e** (cf. Scheme 1 and SM).

**Figure 1:**
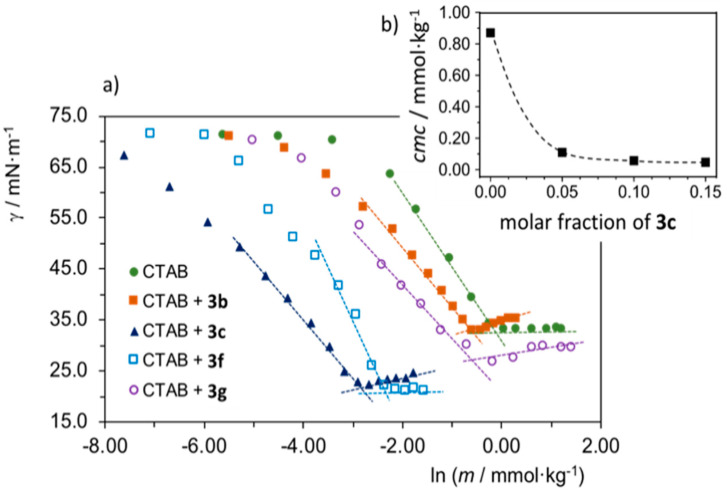
Surface tension plots and *cmc* determination at 25 °C of aqueous surface-active ionic liquid (SAIL)/CTAB mixtures: (**a**) surface tension vs. the logarithm of total SAIL+CTAB concentration expressed in molality; the *cmc* are obtained from the intersection points of the linear fits in each system; (**b**) *cmc* vs. molar fraction of SAIL **3c** in mixtures of SAIL **3c**/CTAB, showing the marked effect of the SAIL in *cmc* reduction.

As mentioned before, compounds **3** display low solubility in water. However, a first indication of their surface activity came from the foaming observed for saturated aqueous solutions of **3c** (cf. Figure S2 of the SM) and, particularly, from surface tension measurements at 25.0 °C, which yielded a value of 29.8 ± 0.3 mN·m^−1^, a considerable decrease from the surface tension of neat water (72.0 mN·m^−1^), hence indicative of strong adsorption of the compound at the air–solution interface. We also verified that the different SAILs **3b**, **3c**, **3f** and **3g** have improved solubility in aqueous solutions of a conventional cationic surfactant, cetyltrimethylammonium bromide (CTAB). For comparisons, we evaluated the effect of adding each SAIL at a fixed 0.10 molar fraction, defined as *x*_SAIL_ = *n*_SAIL_/(*n*_SAIL_ + *n*_CTAB_), on the critical micellar concentration (*cmc*) of the systems and on the surface tension at the *cmc* (*g*_cmc_). The results are shown in Figure 1a and the obtained *cmc* and *g*_cmc_ values are presented in Table 2.

It is clear that all SAILs form mixed micelles with CTAB and induce a marked decrease in *cmc* compared to neat CTAB, even at the low *x*_SAIL_ studied (equal to a SAIL/CTAB molecular ratio of 1:9). Table 2 also shows that there is seemingly a U-shaped variation of *cmc* with an increasing chain length of fatty acids, a somewhat unexpected trend that, on the other hand, demonstrates that surface activities observed are not solely due to the presence of the fatty carboxylate. Significantly, the laurate derivative, **3c**, is the SAIL that brings about the highest decrease in *cmc*, with the mixture’s *cmc* being ca. 15 times smaller than that of neat CTAB. The corresponding *g*_cmc_ for **3c** is also very low, 22 mN·m^−1^, consistent with a strong surface adsorption for this mixture. We further tested the effect of increasing the molar fraction of **3c** on the *cmc* of the mixture (cf. surface tension curves in Figure S3 of the SM). Figure 1b shows that the *cmc* continually decreases with increasing *x*_3c_, again confirming the high interfacial activity of **3c**.

Taken together, these findings not only demonstrate that the **3** RTILs used in the surface activity assays do behave as SAILs, but also suggest that surface activity may influence the antimalarial action of these compounds, as the compound with the most potent antimalarial activity, **3c**, was also the one with a most dramatic effect in lowering *cmc* and surface tension of CTAB, in comparison to the effects exhibited by its counterparts derived from shorter (**3b**) or longer (**3f**,**g**) fatty acids. A more in-depth interpretation of this observation requires further studies, namely by including the butyric (**3a**) and caprylic (**3b**) acid derivatives, among others, in the surface activity assays, in particular because **3a** was also quite active in vitro, despite the fact that butyrate salts are not usually associated with self-assembling properties. Still, the “counterion” to butyrate is, in **3a**, a protonated hydrophobic 4-aminoquinoline, and is hence quite different to counterions used in most common butyrate salts, e.g., sodium butyrate. Moreover, distinct concentration ranges are used in the in vitro assays vs. the surface activity ones, which might put into question the comparability of data from both types of study.

## 3. Concluding Remarks

The observed parallelism between surface activity and antimalarial activity hardly seems coincidental, meaning that this activity, despite being due primarily to the bioactive cation, (i.e., protonated CQ), is also significantly influenced by the amphipathicity and surface activity conveyed by the fatty carboxylate. To the best of our knowledge, this is a first-time disclosure of CQ-derived SAILs whose antimalarial activity is (i) higher than that of CQ, (ii) modulated by the amphipathic anion used, and (iii) influenced by surface activity. Furthermore, the ability of CQ-derived SAILs to co-assemble into colloidal nanostructures (in this case, mixed micelles with CTAB), strongly suggests that these systems could potentially act both as drugs and as enhanced drug delivery systems. We are aware that this may be speculative at this stage, but ongoing studies will shed light onto this and other open questions. Further physico-chemical, biophysical, and biological, including in vivo, studies are under way, which will allow us to fully validate the reported CQ-derived SAILs as a new antimalarial chemotype. Although ILs derived from API have been thoroughly explored over recent years [18,19,20], validation of drug-derived SAILs acting as therapeutic agents that are able to promote their own intracellular delivery will represent a noteworthy development in molecular pharmacology.

## 4. Materials and Methods

### 4.1. Chemical Synthesis

#### 4.1.1. Conversion of Chloroquine Phosphate into **1a**

Commercial chloroquine phosphate was converted into its free base form **1a** as previously described by us for primaquine [11]. Briefly, triethylamine (1.5 mL) was added to a suspension of chloroquine bisphosphate (1.06 g, 2.05 mmol) in dichloromethane (DCM), and the mixture was stirred for 30 min at room temperature (RT). The organic layer was washed with water (10 mL × 3), dried over anhydrous Na_2_SO_4_, and evaporated to dryness under reduced pressure, to afford **1a** (0.63 g, 1.97 mmol) in nearly quantitative yield (93%), and with correct ^1^H-NMR spectral data, as given in the SM.

#### 4.1.2. Synthesis of Chloroquine Analogue **1b**

The synthesis of **1b** was performed as previously described by us [7]. Briefly, 1,4-diaminobutane (1.12 g, 12.7 mmol) and 4,7-dichloroquinoline (0.25 g, 1.27 mmol) were stirred at 100 °C for 3 h. After cooling to RT, the mixture was diluted with DCM (25 mL), and the solution was washed with 5% aqueous Na_2_CO_3_ (25 mL × 3). The organic layer was separated, dried over anhydrous Na_2_SO_4_, filtered, and concentrated to afford **1b** (0.22, 0.87) without need for further purification. Spectroscopic data were in agreement with previous reports [7].

#### 4.1.3. Synthesis of Ionic Liquids **3**

All compounds **3** were synthesized by exactly the same experimental procedure, using the amounts of reactants included in Table S1 of the SM. Briefly, compound **1a** (1 molar equivalent, eq) was dissolved in methanol (10 mL). In parallel, the convenient fatty acid **2a**–**g** (1 eq) was dissolved in methanol (10 mL). The methanolic solution of **1a** was placed under magnetic stirring and the methanolic solution of the convenient fatty acid **2** was added dropwise. Upon addition of the acid, the reaction mixture was kept under stirring for 30 min, at RT. The solvent was removed by evaporation under reduced pressure in the rotary evaporator, and finally dried at high vacuum. The residue obtained was analyzed by ^1^H-NMR and ^13^C-NMR, allowing for verification of the identity of the desired salt, with an anion/cation stoichiometry of 1:1, according to the ^1^H-NMR data given in the SM.

#### 4.1.4. Synthesis of Amides **4**

All compounds **4** were synthesized by exactly the same experimental procedure, using the amounts of reactants included in Table S2 of the SM. Briefly, compound **1b** (1 eq) was dissolved in either dimethylformamide DMF or DCM. In parallel, 1.1 eq of the convenient fatty acid **2a**–**e**, 1.1 eq of *O*-(benzotriazol-1-yl)-*N,N,N′,N′*-tetramethyluronium tetrafluoroborate (TBTU), and 2 eq of *N,N*-diisopropylethylamine (DIEA) were dissolved in DMF or DCM; this solution was placed under magnetic stirring for 10 min at RT, and the solution of **1b** was added dropwise. Stirring was prolonged for 24 h more, at RT in the dark. The mixture was diluted with DCM, and washed 3 times with 5% aqueous Na_2_CO_3_. The organic layer was separated, dried over anhydrous Na_2_SO_4_, and filtered. The solvent was removed under reduced pressure by rotatory evaporation, and the residue was purified by silica gel column chromatography. Chromatographically homogeneous fractions collected were pooled, and the solvent was evaporated under reduced pressure, to afford the final compound. Structural data obtained by ^1^H-NMR, ^13^C-NMR, and ESI-IT MS are given in the SM.

### 4.2. Simultaneous Thermogravimetric Analysis

The thermal stability of the compounds was evaluated using STA equipment from Scancsi, model 7200RV, following the manufacturer’s instructions. The compounds were subjected to heating from room temperature to 500 °C at a speed of 5 °C/min, obtaining the thermograms provided in the SM. For a better visualization of the degradative events, the derivatives of the thermogravimetric curves are also displayed in the thermograms.

### 4.3. Surface Tension Measurements

A DCAT11 tensiometer from Dataphysics GmbH with a Wilhelmy plate was used, and all measurements were performed at 25.0 °C ± 0.5 °C, using a thermostated Julabo F20 circulating bath. The measurements for the *cmc* determination of the CTAB/SAIL solutions were performed by adding aliquots from a stock mixed CTAB/SAIL solution to the solution in the measuring vessel (starting initially with neat water). No dynamic surface tension effects were observed in any measurements.

### 4.4. In Vitro Assays

Laboratory-adapted *Pf* 3D7 (chloroquine- and mefloquine-sensitive), Dd2 (chloroquine-resistant and mefloquine-resistant) were continuously cultured and sorbitol synchronized, as previously described [21]. Staging and parasitemia were determined by light microscopy of Giemsa-stained thin blood smears. Anti-malarial activity was determined using the SYBR Green I assay, as previously described [22]. Briefly, early ring stage parasites (> 80% of rings) were challenged with a 1:3 serial dilution in medium from a stock solution of each compound in DMSO, with final concentrations ranging from 10,000–0.169 nM. Fluorescence intensity was measured with a multi-mode microplate reader (Triad, Dynex Technologies), with excitation and emission wavelengths of 485 and 535 nm, respectively, and analyzed by nonlinear regression using GraphPad Prism to determine IC_50_ values.

## Figures and Tables

**Table 1:** Synthesis yields, thermal degradation data, and in vitro activity against *Plasmodium falciparum* (*Pf*) 3D7 and Dd2 strains obtained for room temperature ionic liquids (RTILs) **3a**–**e** and for their covalent analogues **4a**–**e**; thermal degradation data also provided for CQ phosphate and **2a**–**e**; the in vitro activity data obtained for the commercial CQ phosphate salt, for **2c**, and for an equimolar mixture of these two compounds are also included.

Compound	Synthesis Yield/%	Temperature of Degradation Events Observed/°C	Half-Maximal Inhibitory Concentration (IC_50_) ± SD/nM	
			*Pf* 3D7	*Pf* Dd2
**3a**	99	93.2; 236.9	12 ± 5	384 ± 142
**3b**	99	120.7; 220.5	13 ± 2	402 ± 190
**3c**	88	156.9; 228.5	4 ± 1	110 ± 36
**3d**	98	227.7	12 ± 5	235 ± 79
**3e**	99	197.1	15 ± 4	365 ± 126
**4a**	75	291.3	627 ± 142	588 ± 44
**4b**	66	314.1	51 ± 9	109 ± 7
**4c**	74	310.8	70 ± 7	160 ± 14
**4d**	70	335.2	n.d. ^2^	n.d. ^2^
**4e**	48	336.5	n.d. ^2^	n.d. ^2^
**2a**	−	75.5	n.d.	n.d.
**2b**	−	131.0	n.d.	n.d.
**2c**	−	172.3	>10,000	>10,000
**2d**	−	218.5	n.d.	n.d.
**2e**	−	211.6	n.d.	n.d.
**CQ ^1^**	−	301.7	45 ± 15	660 ± 11
**CQ ^1^ + 2c**	−	−	59 ± 16	415 ± 44

^1^ chloroquine phosphate (standard formulation of this API); ^2^ insoluble; n.d., not determined.

**Table 2:** Critical micellar concentration (*cmc*) and surface tension at the *cmc* (©_cmc_) for CTAB and different CTAB/SAIL solutions with a molar fraction of SAIL, x_SAIL_, equal to 0.10.

System	cmc/mmol·kg^−1^	©_cmc_/mN·m^−1^
CTAB	0.84	33.0
CTAB + **3b**	0.57	32.4
CTAB + **3c**	0.057	22.0
CTAB + **3f**	0.10	20.8
CTAB + **3g**	0.40	30.0

## Supplementary Materials

The following are available online at https://www.mdpi.com/1422-0067/21/15/5334/s1, Table S1: amounts of reactants used for the synthesis of **3a–g**; spectral data and traces for compounds **3a–g**; Table S2: amounts of reactants used for the synthesis of **4a–e**; spectral data and traces for compounds **4a–e**; Figure S1: superimposed ^1^H NMR spectra of octanoic acid **2b**, basic chloroquine **1a**, and their derived ionic liquid **3b**; thermograms for ionic liquids **3**; thermograms for amides **4**; Figure S2: appearance of a saturated solution of **3c** in water, displaying turbidity and foam formation; Figure S3: surface tension plots and cmc determination, at 25.0 °C, of aqueous CTAB/SAIL **3c** mixtures for increasing molar fraction of **3c**; Table S4: values for cmc and surface tension at the cmc (γcmc) for CTAB/SAIL 3c with increasing molar fraction of **3c**.

## Abbreviations

API	Active pharmaceutical ingredient
CQ	Chloroquine
CTAB	Cetyltrimethylammonium bromide
DCM	Dichloromethane
DIEA	*N*-ethyl-*N*,*N*-diisopropylamine
DMF	Dimethylformamide
DMSO	Dimethylsulfoxide
DMSO-d6	Hexadeuterated dimethylsulfoxide
eq	Molar equivalent
ESI-IT MS	Electrospray ionization-ion trap mass spectrometry
IC_50_	Half-maximal inhibitory concentration
IL	Ionic liquid
MeOH	Methanol
NMR	Nuclear magnetic resonance
*Pf*	*Plasmodium falciparum*
PQ	Primaquine
RT	Room temperature
RTIL	Room temperature ionic liquid
SAIL	Surface-active ionic liquid
SD	Standard deviation
SM	Supplementary materials
STA	Simultaneous thermogravimetric analysis
TBTU	*O*-(benzotriazol-1-yl)-*N,N,N′,N*′-tetramethyluronium tetrafluoroborate
