# Supplementary material for: Building on Surface-Active Ionic Liquids for the Rescuing of the Antimalarial Drug Chloroquine
Source: Int J Mol Sci. 2020 Jul 27;21(15):5334. doi: 10.3390/ijms21155334 (PMC7432003; doi:10.3390/ijms21155334)
Supplement: Supplementary file 1 [file ijms-21-05334-s001.pdf]

International Journal of Molecular Sciences

MDPI

Communication

Building on Surface-Active Ionic Liquids for the Rescuing of the Antimalarial Drug Chloroquine

Ana Teresa Silva [1], Lis Lobo [2], Isabel S. Oliveira [3], Joana Gomes [1,3], Cátia Teixeira [1], Fátima Nogueira [2], Eduardo F. Marques [3], Ricardo Ferraz [1,4] and Paula Gomes [1,*]

[1] LAQV-REQUIMTE, Departamento de Química e Bioquímica, Faculdade de Ciências, Universidade do Porto, P-4169-007 Porto, Portugal; up201303026@gmail.com (A.T.S.); up201606401@fc.up.pt (J.G.); catia.teixeira@fc.up.pt (C.T.); pgomes@fc.up.pt (P.G.); ricardoferraz@eu.ipp.pt (R.F.)

[2] Global Health and Tropical Medicine, Instituto de Higiene e Medicina Tropical, Universidade Nova de Lisboa, P-1349-008 Lisboa, Portugal; lis.lobo@ihmt.unl.pt (L.L.); FNogueira@ihmt.unl.pt (F.N.)

[3] CIQ-UP, Departamento de Química e Bioquímica, Faculdade de Ciências, Universidade do Porto, P-4169-007 Porto, Portugal; isabelmscoliveira@gmail.com (I.S.O.); efmarque@fc.up.pt (E.F.M.)

[4] Ciências Químicas e das Biomoléculas, Escola Superior de Saúde, Politécnico do Porto, P-4200-072 Porto, Portugal

* Correspondence: pgomes@fc.up.pt; Tel.: +351 220402563

Received: 10 July 2020; Accepted: 24 July 2020; Published: date

1. Synthesis and spectral data

1.1. *chloroquine (free base), 1a*

1a Colorless oil; δ_H (DMSO-d6, 400 MHz) 8.35 (m, 2H, Q3 and Q8), 7.76 (d, 1H, J = 2.2Hz, Q5), 7.42 (dd, 1H, J = 9.0, 2.3Hz, Q7), 6.90 (d, J = 8.0Hz, -NH-), 6.50 (d, 1H, J = 5.7, Q2), 3.71 (m, 1H, CQ1), 2.40 (q, 4H, CQ5 and CQ7), 2.36 (t, 2H, J = 6.9Hz, CQ4), 1.55 (m, 4H, CQ2 and CQ3), 1.23 (d, 3H, J = 6.4Hz, $-CH_3$), 0.91 (d, 6H, J = 7.1Hz, CQ8 and CQ6).

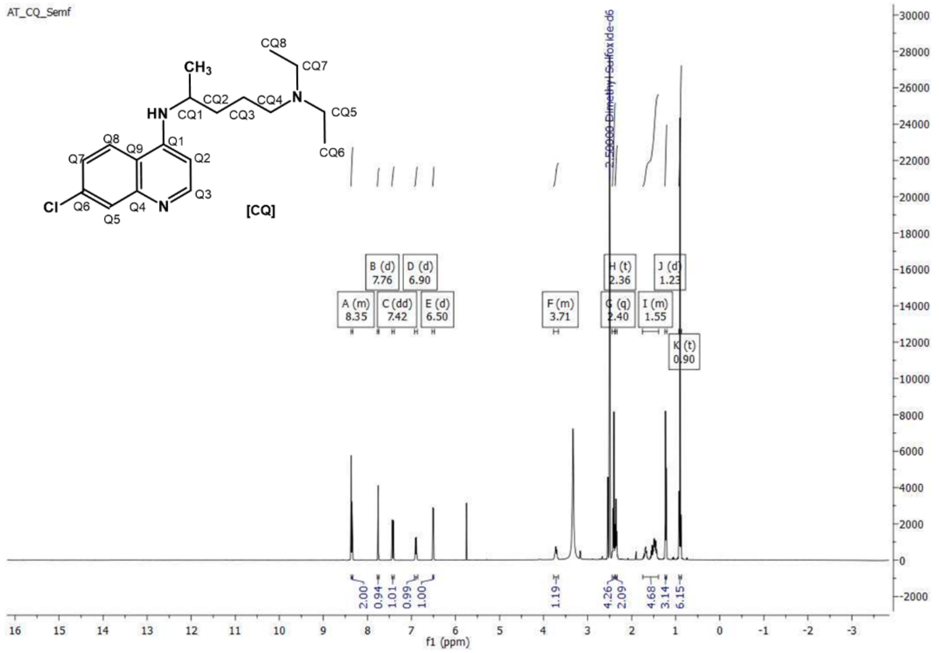

^{1}H-NMR spectrum of 1a (400 MHz, DMSO-d6).

1.2. *Ionic liquids 3*

a) Amounts of reactants used for the synthesis of 3a-g

Table S1.

Target Product	Chloroquine (1a) / mg (mmol)	Fatty Acid (2a-g) / mg (mmol)
**3a**	630 (1.97)	174 (1.97)
**3b**	576 (1.80)	260 (1.20)
**3c**	285 (0.89)	174 (0.87)
**3d**	275 (0.86)	245 (0.86)
**3e**	256 (0.80)	226 (0.80)
**3f**	376 (1.20)	264 (1.20)
**3g**	288 (0.90)	231 (0.90)

b) Spectral data and traces for compounds 3a-g

3a, Colorless oil; **δ$_H$** (DMSO-d6, 400 MHz) 8.37 (m, 2H, Q3 and Q8), 7.76 (d, 1H, *J* = 2.2Hz, Q5), 7.42 (dd, 1H, *J* = 9.0, 2.3Hz, Q7), 6.90 (d, 1H, *J* = 8.1Hz, -NH-), 6.50 (d, 1H, *J* = 5.7Hz, Q2), 3.71 (m, 1H, CQ1), 2.42 (q, 4H, *J* = 7.1Hz, CQ5 and CQ7), 2.36 (t, 2H, *J* = 7.0Hz, CQ4), 2.16 (t, 2H, *J* = 7.3Hz, A2), 1.70 (m, 2H, CQ2), 1.50 (m, 4H, CQ3 and A3), 1.23 (d, 3H, *J* = 6.4Hz, CH_3), 0.90 (t, 6H, *J* = 6.4Hz, CQ6 and CQ8), 0.87 (t, 3H, *J* = 6.7Hz, A4); **δ$_C$** (DMSO-d$_6$, 100 MHz) 174.89 (A1), 152.34 (Q3), 150.00 (Q1), 149.76 (Q4), 133.79 (Q6), 127.88 (Q5), 124.82 (Q8), 124.23 (Q7), 117.97 (Q9), 99.30 (Q2), 52.52 (CQ4), 48.05 (CQ1), 46.63 (CQ5 and CQ7), 36.19 (A2), 33.83 (CQ2), 23.81 (-CH_3), 20.31 (A3), 18.44 (CQ3), 18.44 (A4), 12.04 (CQ6 and CQ8); **ESI-IT MS (+)** ($C_{18}H_{27}ClN_3^+$, 320.19 a.m.u.) $^m/_z$: 320.60 a.m.u. (MH^+).

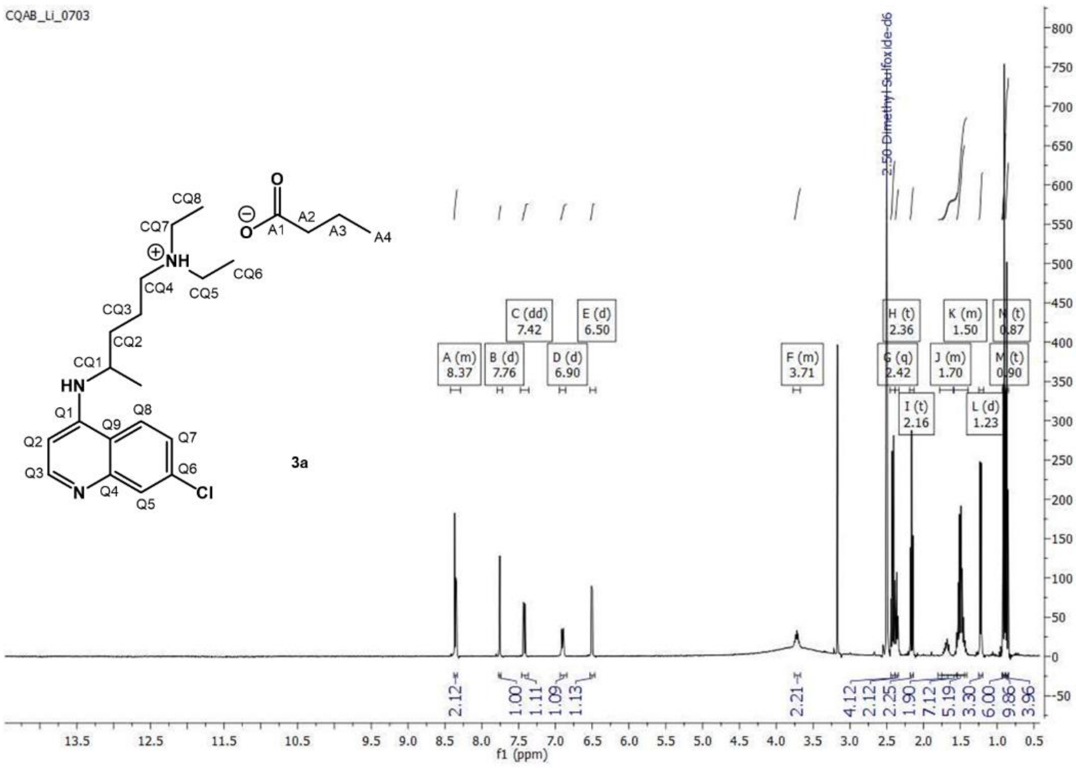

^{1}H-NMR spectrum of 3a (400 MHz, DMSO-d$_6$).

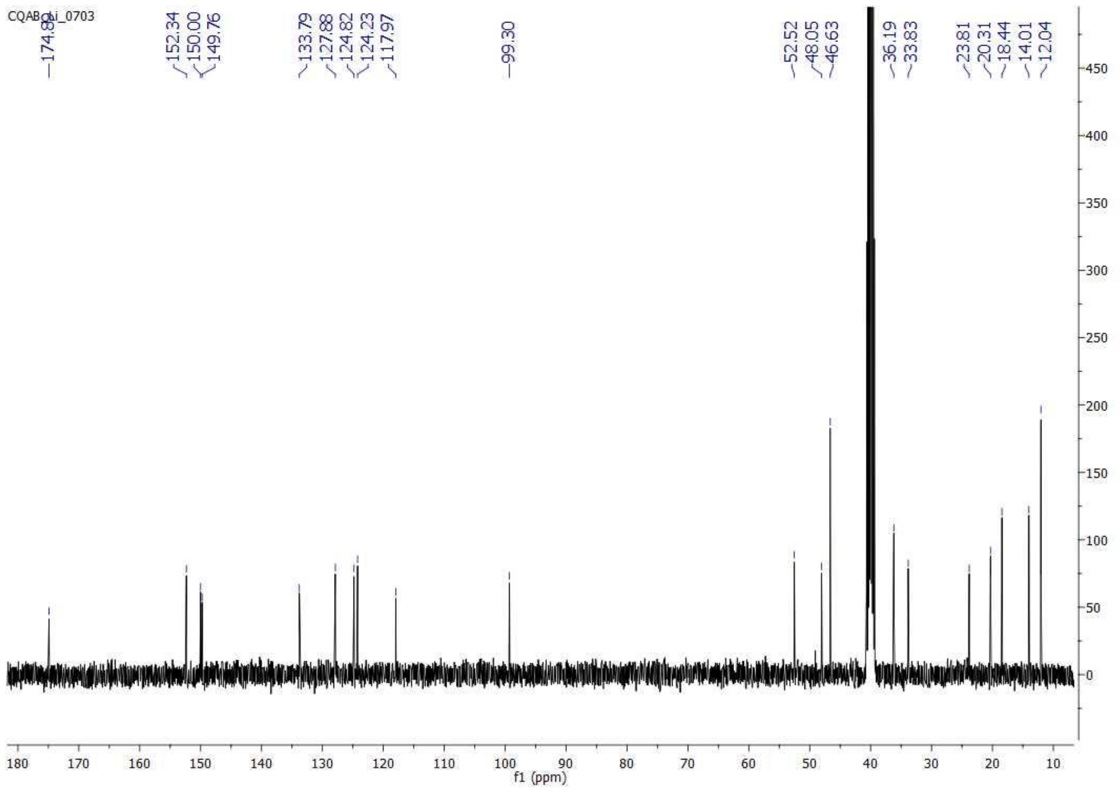

^{13}C-NMR spectrum of 3a (100 MHz, DMSO-d$_6$).

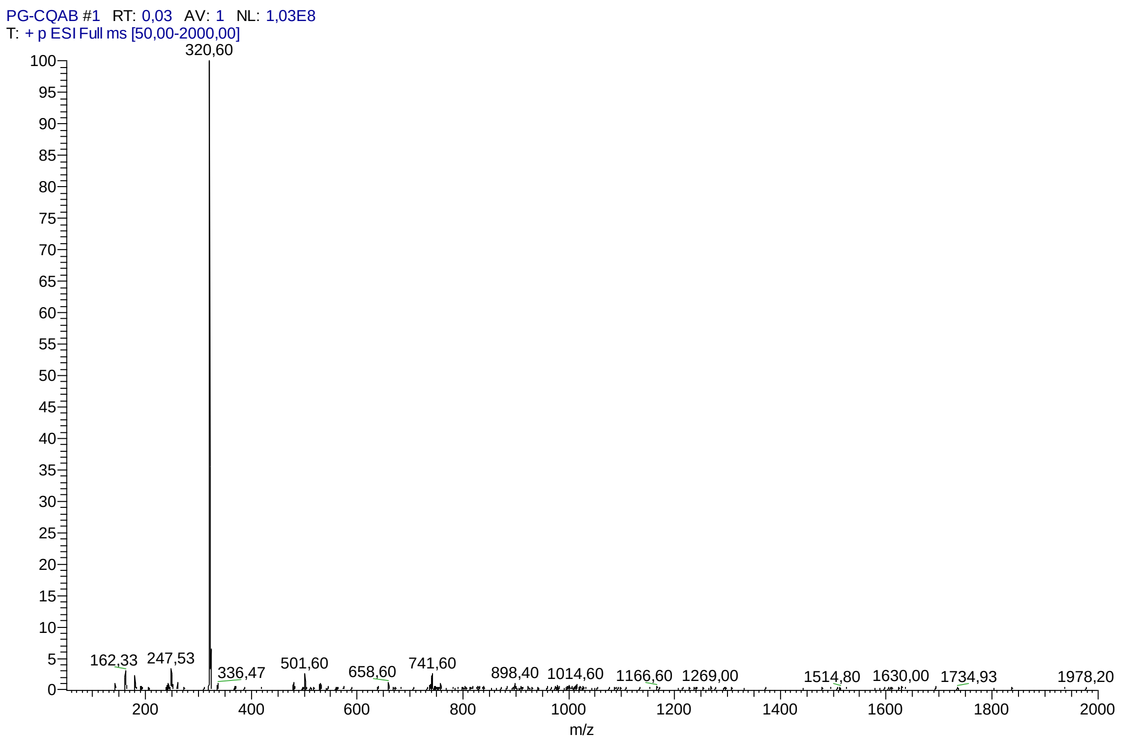

ESI-IT mass spectrum for 3a (positive mode).

3b, Colorless oil; δ_H (DMSO-d6, 400 MHz) 8.36 (m, 2H, Q3 and Q8), 7.76 (d, 1H, *J* = 2.2Hz, Q5), 7.42 (dd, 1H, *J* = 9.0, 2.0Hz, Q7), 6.90 (d, 1H, *J* = 7.7Hz, -NH-), 6.51 (d, 1H, *J* = 5.5Hz, Q2), 3.68 (m, 1H, CQ1), 2.40 (q, 4H, *J* = 7.1Hz, CQ5 and CQ7), 2.36 (t, 2H, *J* = 6.9Hz, CQ4), 2.17 (t, 2H, *J* = 7.4Hz, A2), 1.70 (m, 2H,CQ2), 1.50 (m, 4H, CQ3 and A3), 1.23 (s, 11H, -CH$_2$- and -CH$_3$), 0.90 (t, 6H, *J* = 7.1Hz, CQ6

and CQ8), 0.85 (t, 3H, *J* = 6.9Hz, A8); δ_C ($CDCl_3$, 100 MHz) 179.65 (A1), 150.64 (Q1), 150.36 (Q4), 148.03(Q3), 136.23 (Q6), 127.11 (Q5), 125.11(Q8), 122.68 (Q7), 117.35 (Q9), 98.69 (Q2), 51.65 (CQ1), 48.40 (CQ4), 45.93 (CQ5 and CQ7), 36.85 (A2), 33.54 (CQ2), 31.75 (A6), 29.54(A5), 29.12 (A4), 26.04 (A3), 22.60 (A7), 22.12 (-CH_3), 20,09 (CQ3), 14.05 (A8), 9.31 (CQ6 and CQ8); **ESI-IT MS (+)** ($C_{18}H_{27}ClN_3^+$, 320.19 a.m.u.) $^m/_z$: 320.60 a.m.u. (MH^+).

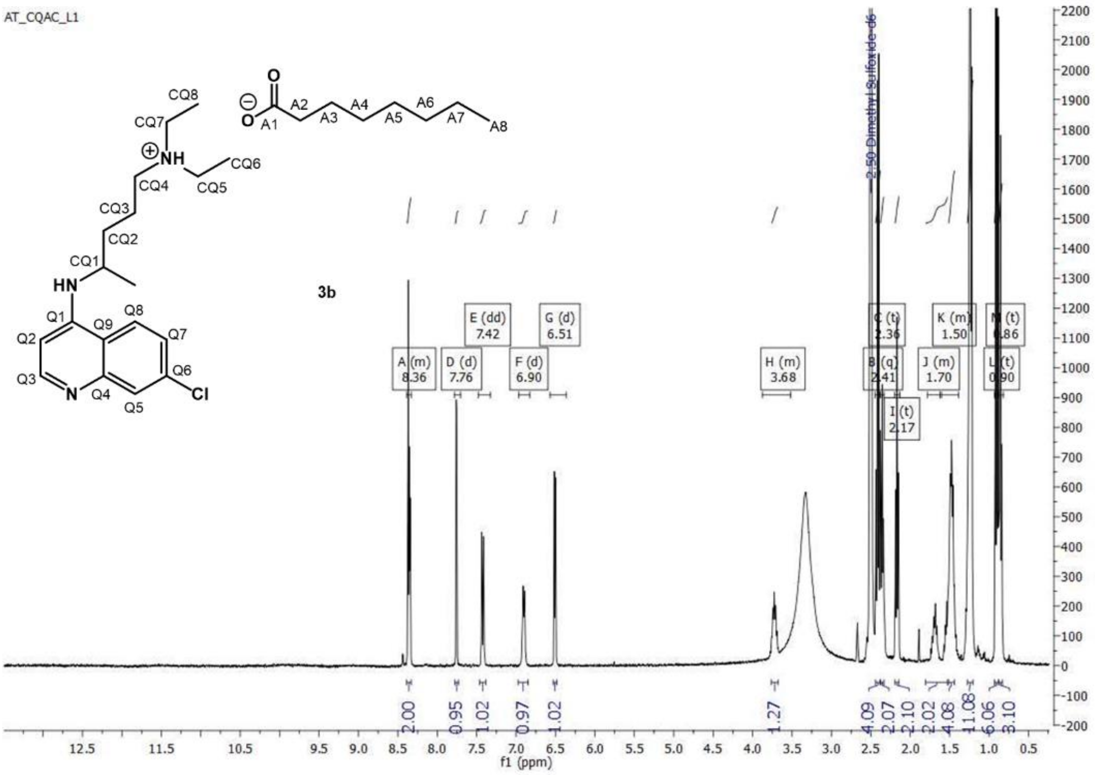

^1H-NMR spectrum of 3b (400 MHz, DMSO-d_6).

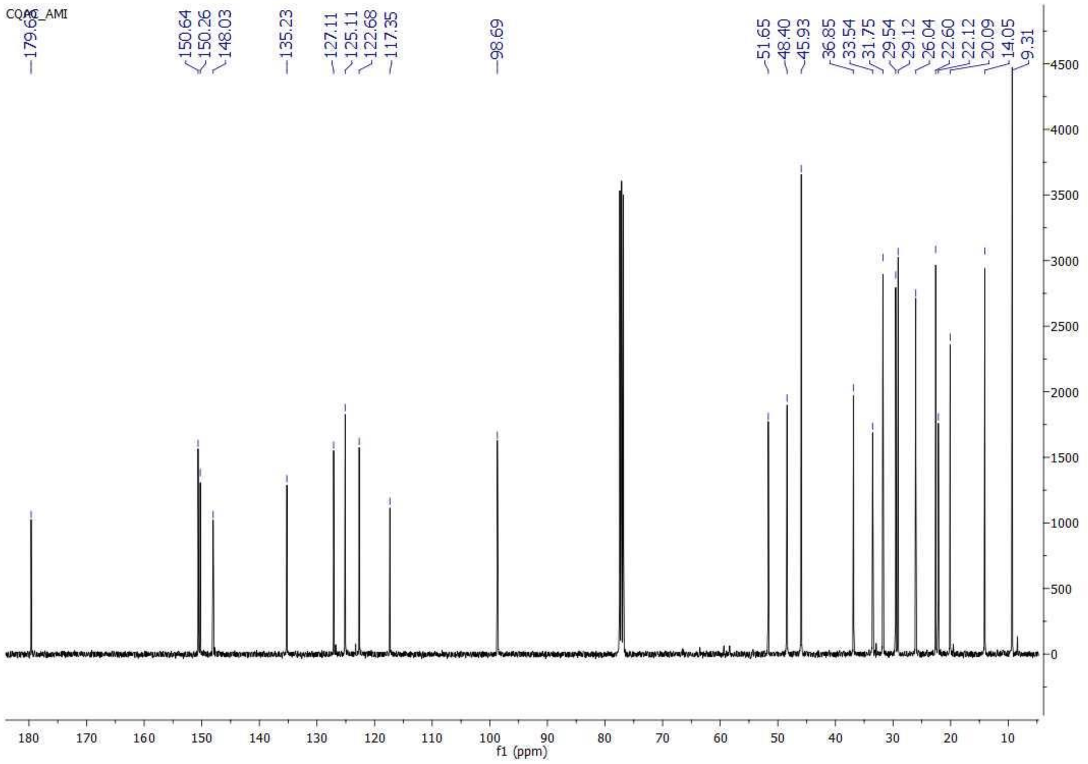

^{13}C-NMR spectrum of 3b (100 MHz, $CDCl_3$).

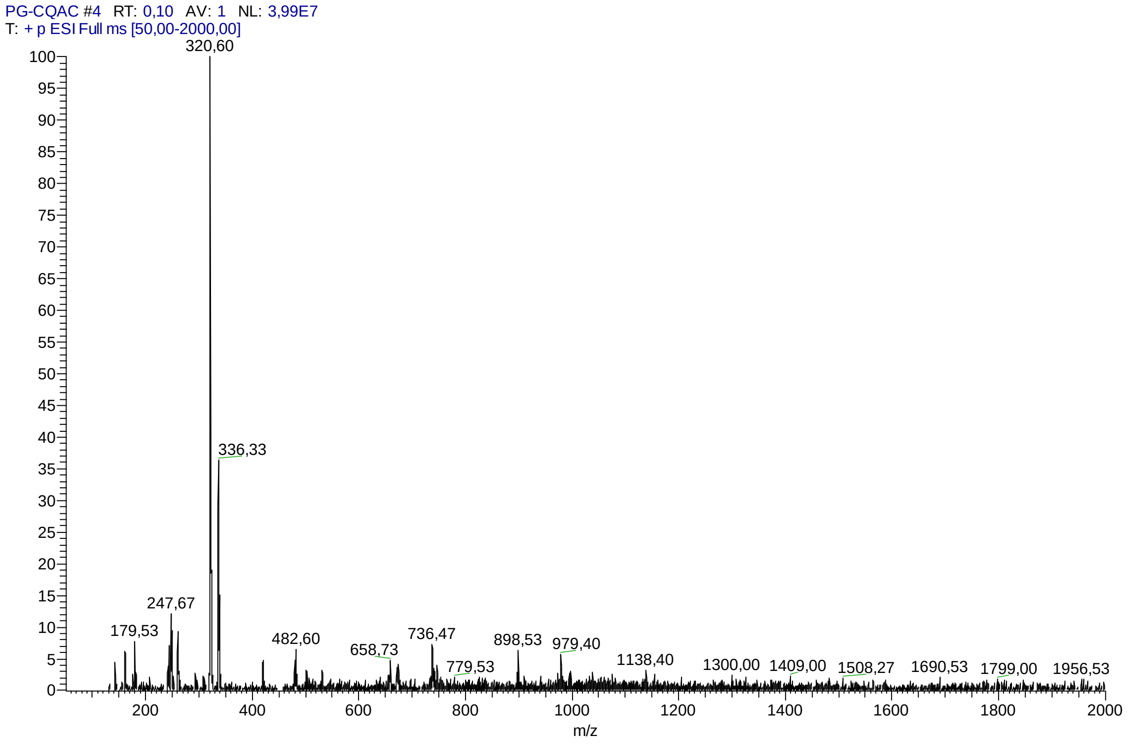

ESI-IT mass spectrum for 3b (positive mode).

3c, Colorless oil; **δ$_H$** (DMSO-d6, 400 MHz) 8.35 (m, 2H, Q3 and Q8), 7.76 (d, 1H, *J* =2.2Hz, Q5), 7.42 (dd, 1H, *J*= 9.0, 2.3Hz, Q7), 6.90 (d, 1H, *J*= 8.2Hz, -NH-), 6.51(d, 1H, *J*= 5.7Hz, Q2), 3.67 (m, 1H, CQ1), 2.42 (q, 4H, *J*= 7.1Hz, CQ5 and CQ7), 2.37 (t, 2H, *J*=6.9Hz, CQ4), 2.17 (t, 2H, *J* = 7.4Hz, A2), 1.69 (m, 2H, CQ2), 1.51 (m, 4H, CQ3 and A3), 1,22 (s, 31H, -CH_2- and -CH_3), 0,90 (t, 6H, *J*= 7.1Hz, CQ6 and CQ8), 0,85 (t, 3H, *J*= 6.9Hz, A12); **δ$_C$** ($CDCl_3$, 100 MHz) 179.59 (A1), 150.91 (Q1), 150.07 (Q4), 148.30 (Q3), 135.21 (Q6), 127.45 (Q5), 125.16 (Q8), 122.44 (Q7), 117.37 (Q9), 98.77 (Q2), 51.73 (CQ4), 48.38 (CQ1), 46.00 (CQ5 and CQ7), 36.79(A2), 33.61 (CQ2), 31.90 (A10), 29.65 (A6), 29.62 (A7 and A8), 29.59 (A9), 29.51 (A5), 29.33 (A4), 26.04 (A3), 22.67 (A11), 22.21 (-CH_3), 20,16 (CQ3), 14.10 (A12), 11.78 (CQ8 and CQ6); **ESI-IT MS (+)** ($C_{18}H_{27}ClN_3^+$, 320.19 a.m.u.) $^m/_z$: 320.60 a.m.u. (MH^+).

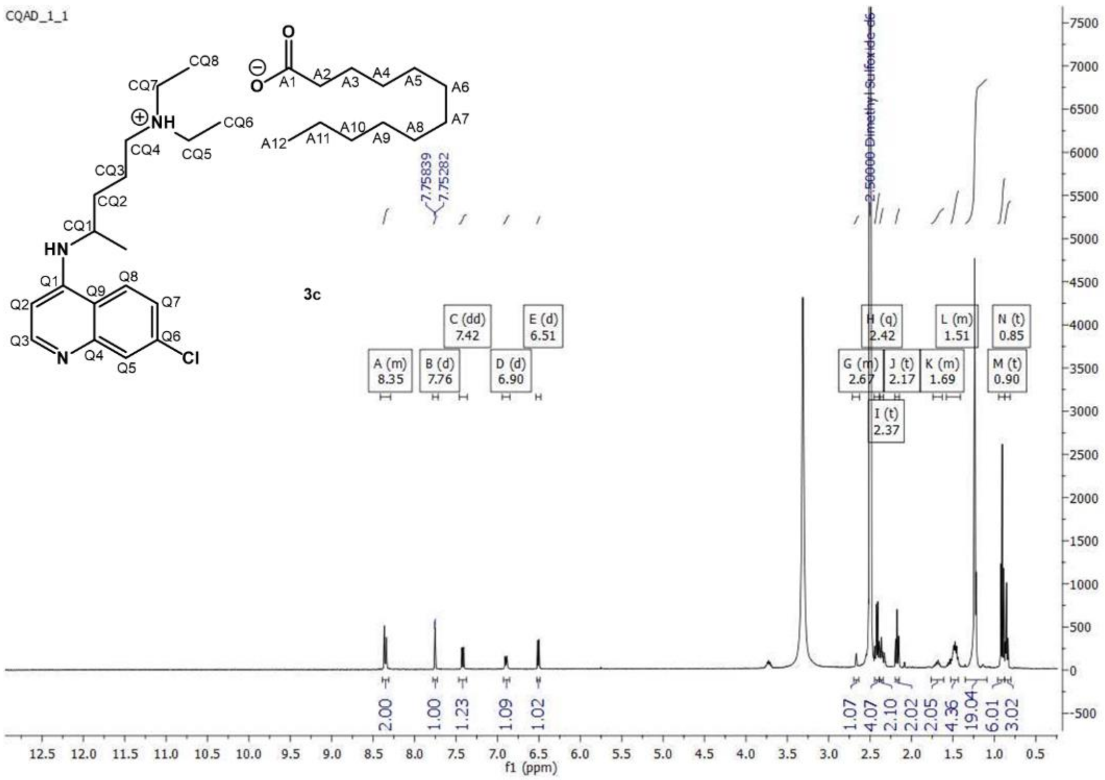

^{1}H-NMR spectrum of 3c (400 MHz, DMSO-d_6).

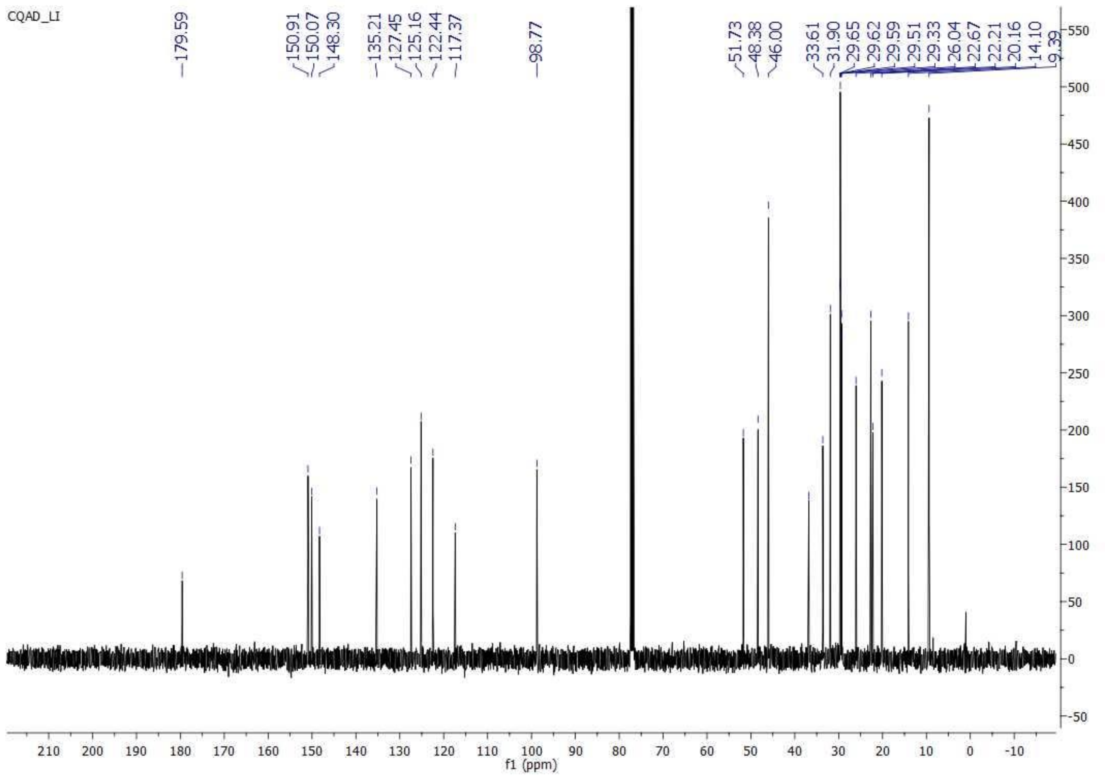

^{13}C-NMR spectrum of 3c (100 MHz, $CDCl_3$).

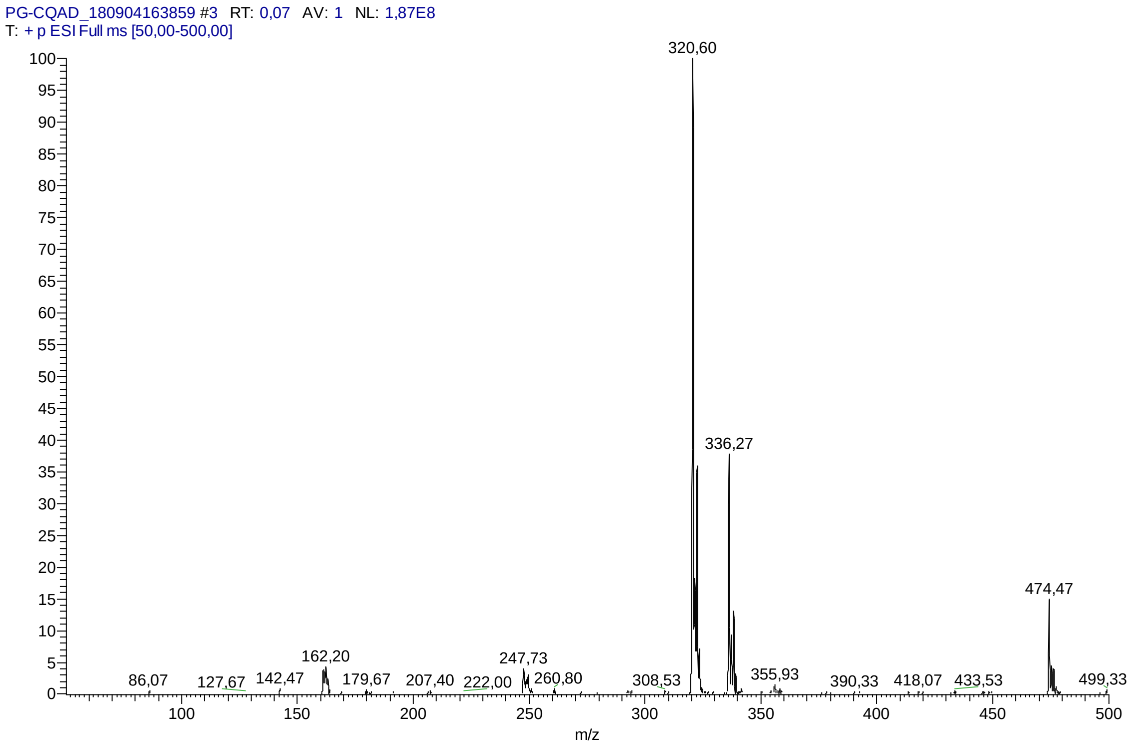

ESI-IT mass spectrum for 3c (positive mode).

3d, yellow oil; **δ_H** (DMSO-d6, 400 MHz) 8.34 (m, 2H, Q3 and Q8), 7.76 (d, 1H, *J* =2.2Hz, Q5), 7.41 (dd, 1H, *J*= 9.0, 2.2Hz, Q7), 6.92(d, 1H, *J*= 7.9Hz, -NH-), 6.51 (d, 1H, *J*= 5.6Hz, Q2), 3.70 (m,1H, CQ1), 2.45 (q, 4H, *J*= 14.3, 7.2Hz, CQ5 and CQ7), 2.40 (t, 2H, *J*= 7.0Hz, CQ4), 2.16 (t, 2H, *J*= 7.3Hz, A2), 1.68 (m, 2H, CQ2), 1.48 (m, 4H, CQ3 and A3), 1.22 (s, 31H, $-CH_2-$ and $-CH_3-$), 0.91 (t, 6H, *J*= 7.1Hz, CQ6 and CQ8), 0.84 (t, 3H, *J*= 6.8Hz, A18); **δ_C** (DMSO-d6, 100 MHz) 175.10 (A1), 152.30 (Q3), 150.07 (Q1), 149.64 (Q4), 133.06 (Q6), 127.77 (Q5), 124.79 (Q8), 124.29 (Q7), 117.94 (Q9), 99.30 (Q2), 52.41 (CQ4), 48.05 (CQ1), 46.62 (CQ5 and CQ7), 34.38 (A2), 33.75 (CQ2), 31.74 (A16), 29.45 (A6-13), 29.35 (A14), 29.19 (A4), 29.14 (A15), 20.01 (A5), 25.01 (A3), 23.56 (A17), 22.54 ($-CH_3$), 20.29 (CQ3), 14.40 (A18), 11.78 (CQ8 and CQ6); **ESI-IT MS (+)** ($C_{18}H_{27}ClN_3^+$, 320.19 a.m.u.) $^m/_z$: 320.60 a.m.u. (MH^+).

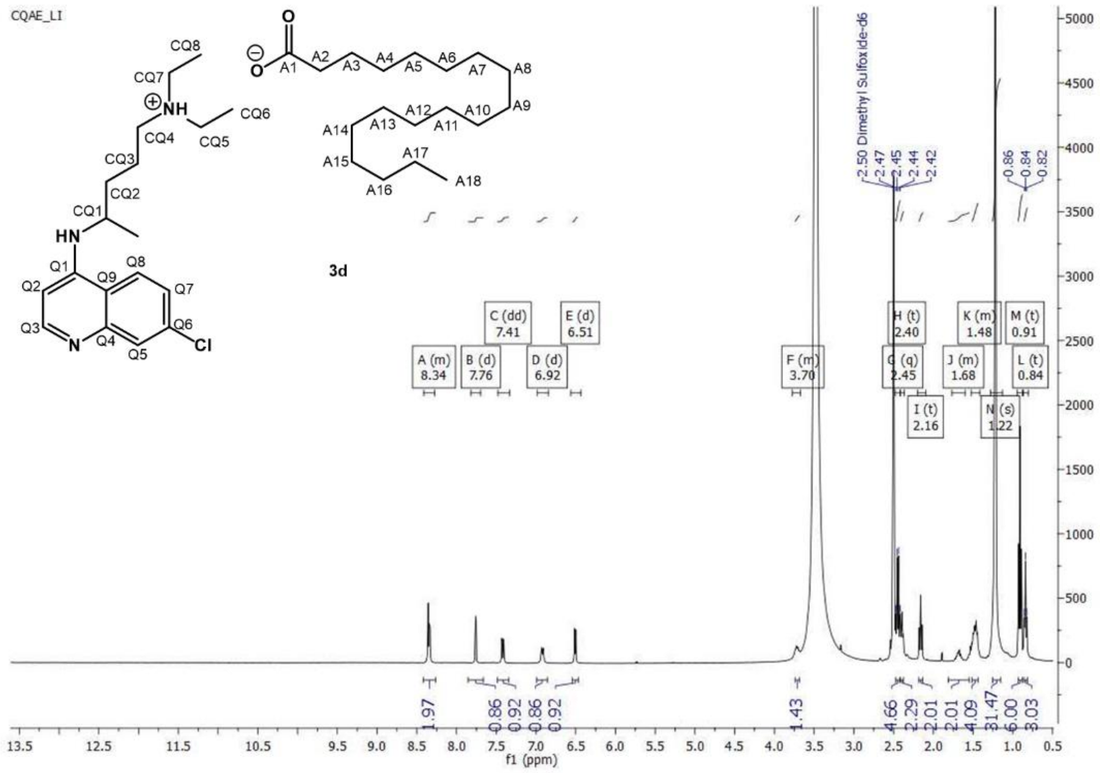

^{1}H-NMR spectrum 3d (400 MHz, DMSO-d6).

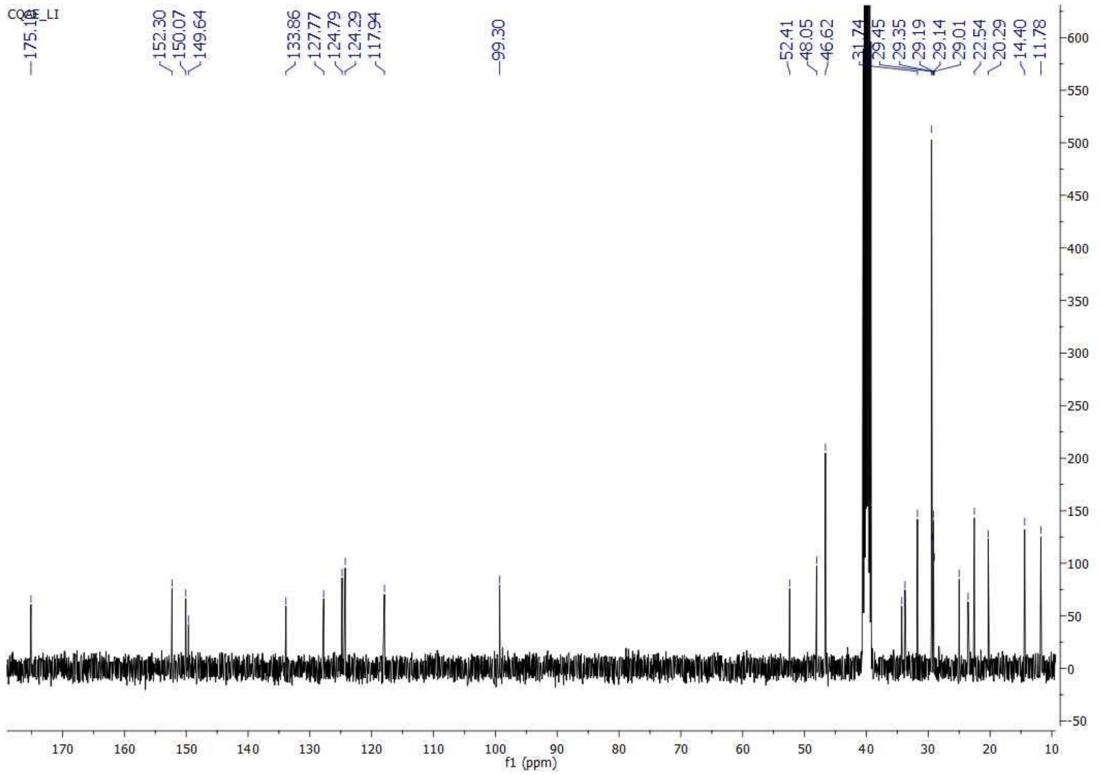

^{13}C-NMR spectrum of 3d (100 MHz, DMSO-d6).

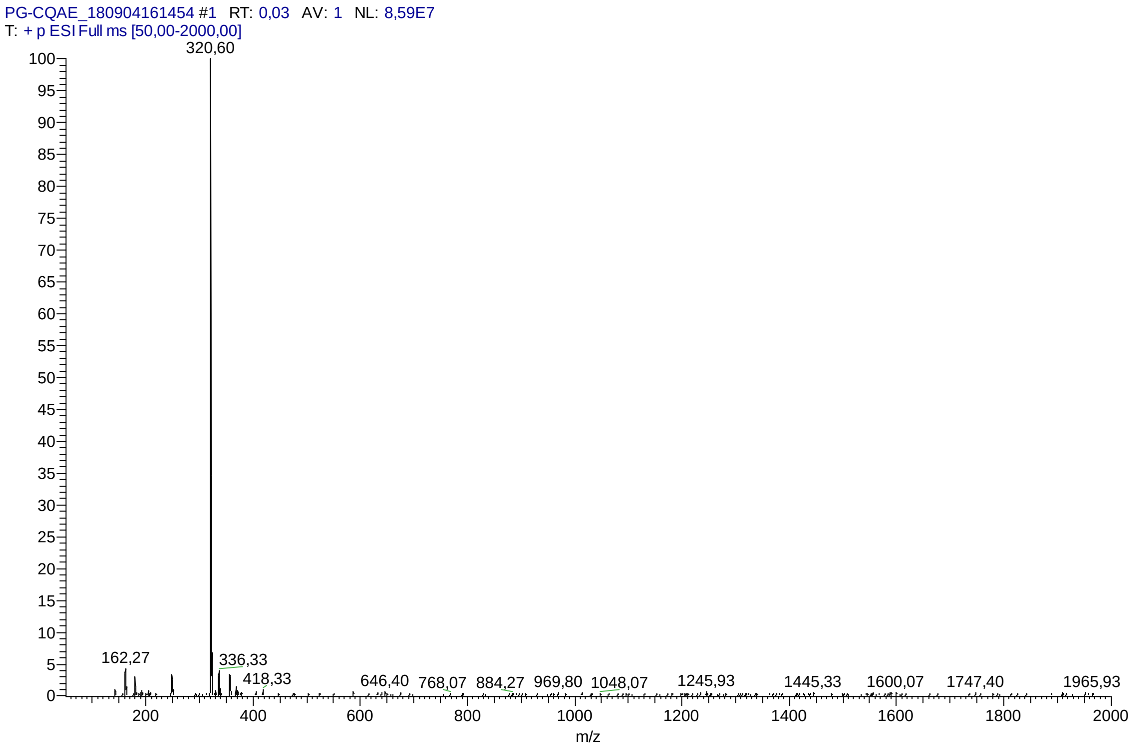

ESI-IT mass spectrum for 3d (positive mode).

3e, yellow oil; **δ_H** (DMSO-d6, 400 MHz) 8.35 (m, 2H, Q3 and Q8), 7.76 (d, 1H, *J*= 2.2Hz, Q5), 7.42 (dd, 1H, *J*= 9.0, 2.3Hz, Q7), 6.90 (d, 1H, *J*= 8.1Hz, -NH-), 6.51 (d, 1H, *J*= 5.7Hz, Q2), 5.32 (t, 2H, Ha e Hb), 3.72 (m, 1H, CQ1), 2.41 (q, 4H, *J*= 7.1Hz, CQ5 and CQ7), 2.36 (t, 2H, *J*= 7.0Hz, CQ4), 2.16 (t, 2H, *J*= 7.4 Hz, A2), 1.98 (q, 4H, A8 and A11), 1.70 (m, 2H, CQ2), 1.46 (m, 4H, CQ3 and A3), 1.22 (s, 23H, -CH_2- and -CH_3), 0.90 (t, 6H, *J*= 7.1Hz, CQ6 and CQ8), 0.84 (t, 3H, *J*= 6.8Hz, A18); **δ_C** ($CDCl_3$, 100 MHz) 179.34 (A1), 150.53 (Q1), 150.36 (Q4), 147.87 (Q3), 135.47 (Q6), 129.90 (A9), 129.85 (A10), 127.09 (Q5), 125.32 (Q8), 122.64 (Q7), 117.32 (Q9), 98.66 (Q2), 51.65 (CQ4), 48.45 (CQ1), 45.91 (CQ5 and CQ7), 36.51 (A2), 33.41 (CQ2), 31.90 (A16), 29.77 (A7 and A12), 29.70 (A6), 29.56 (A13), 29.52 (A14), 29.41 (A8), 29.31 (A11), 29.26 (A5), 27.22 (A4 and A15), 25.93 (A3), 22.68 (A17), 21.92 (-CH_3), 20.20 (CQ3), 14.12 (A18), 9.02 (CQ8 and CQ6); **ESI-IT MS (+)** ($C_{18}H_{27}ClN_3^+$, 320.19 a.m.u.) $^m/_z$: 320.53 a.m.u. (MH^+).

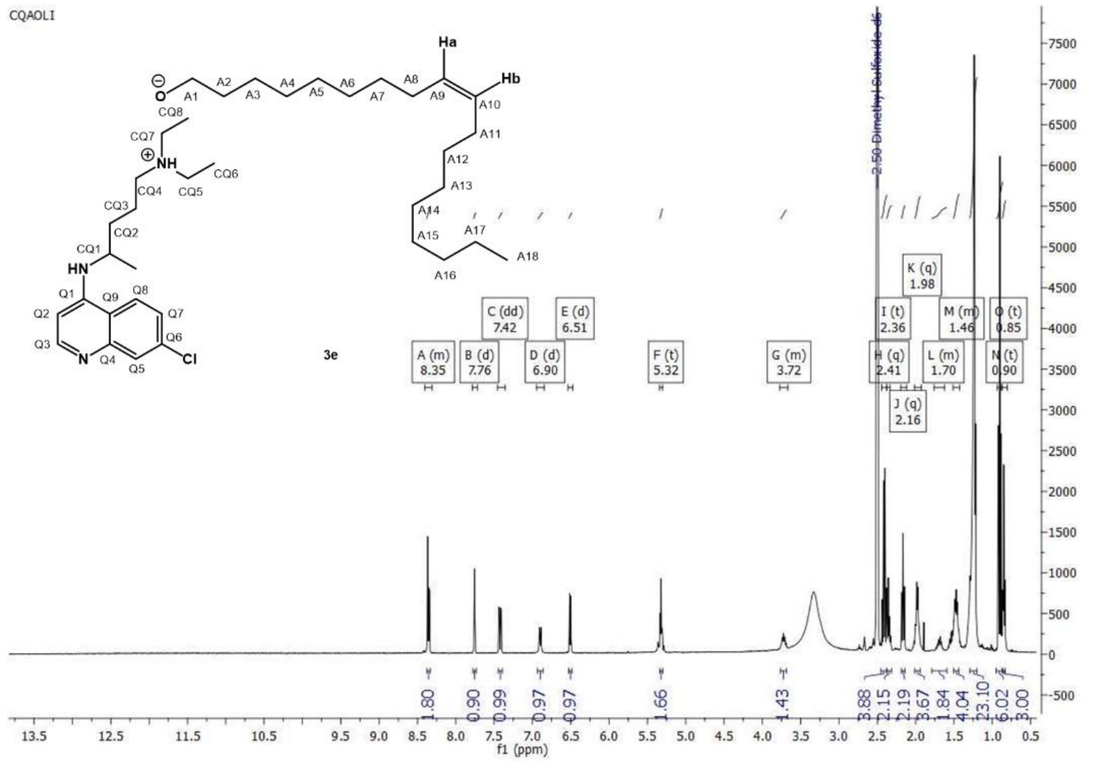

^{1}H-NMR spectrum of 3e (400 MHz, DMSO-d_6).

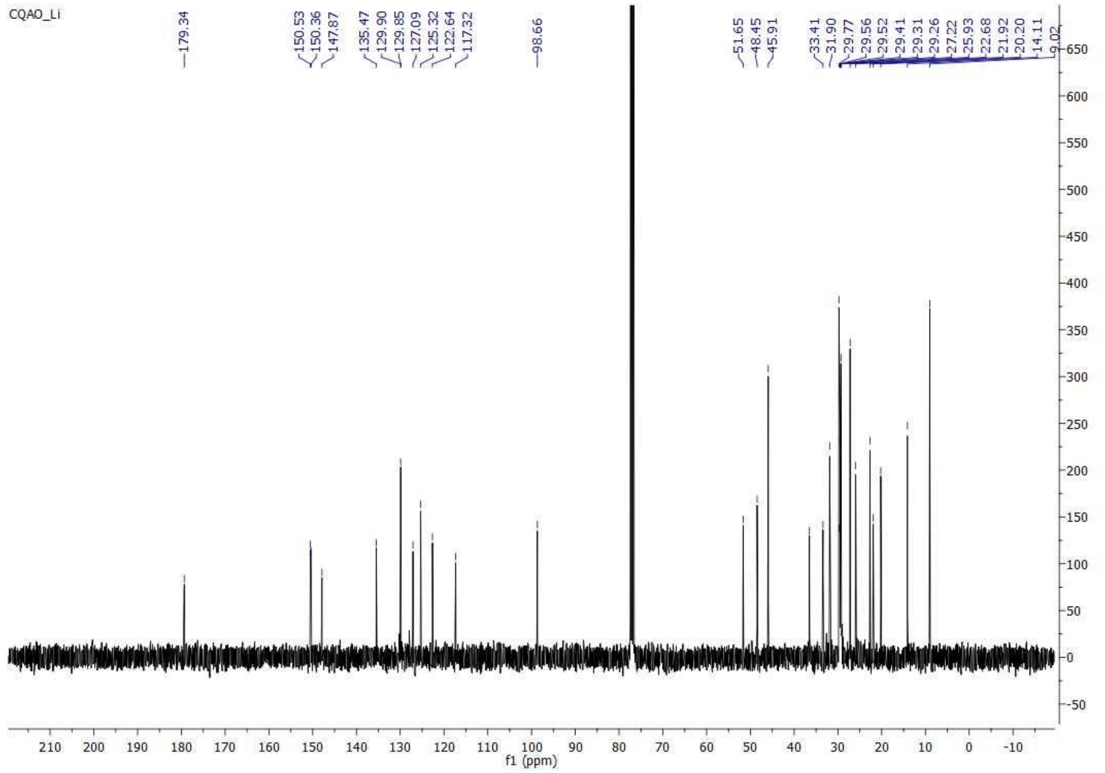

^{13}C-NMR spectrum of 3e (100 MHz, $CDCl_3$).

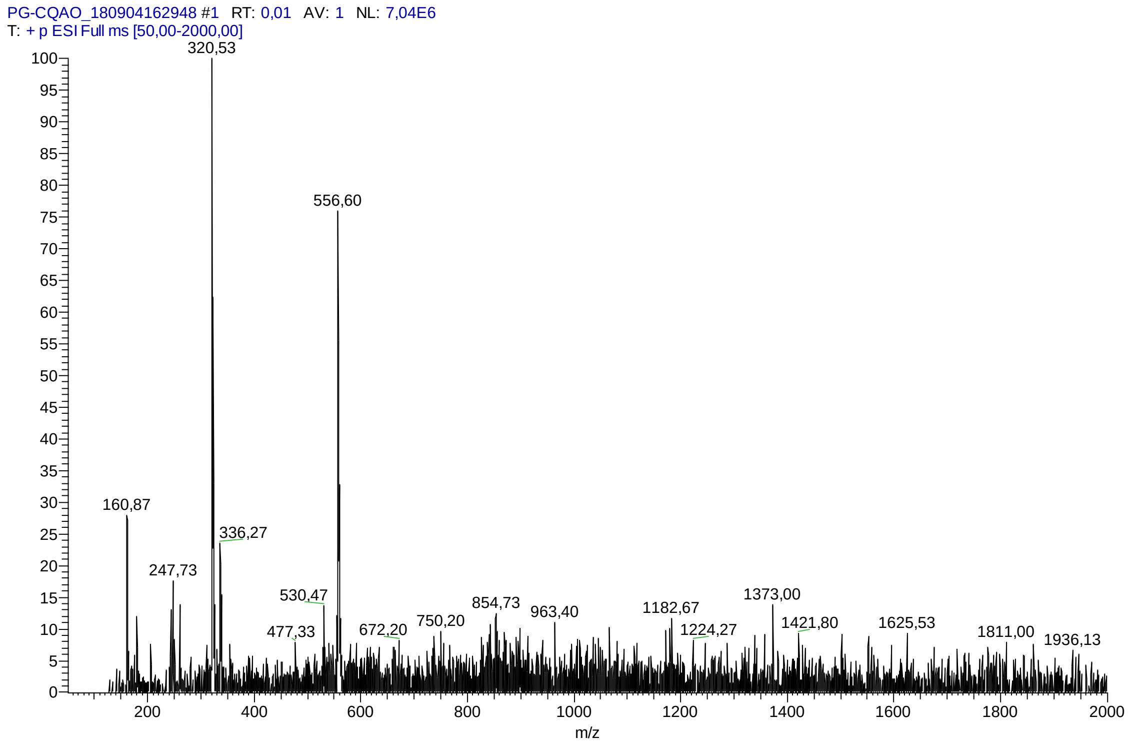

ESI-IT mass spectrum for 3e (positive mode).

3f, yellow oil; **δ**$_H$ ($CDCl_3$, 400 MHz) 8.51 (d, 1H, *J*=5.5Hz, Q3), 7.79 (d, 1H, *J*=8.96Hz, Q8), 7.96 (d, 1H, *J*= 2.13Hz, Q5), 7.35 (dd, 1H, *J*= 9.0, 2.2Hz, Q7), 6.39 (d, 1H, *J*= 5.73Hz, Q2), 5.89 (s, 1H, -NH-), 1.12 (t, 6H, *J*= 7.24Hz, CQ6 and CQ8), 0.87 (t, 3H, *J*= 6.87Hz, A18); **δ**$_C$ (DMSO-d6, 100 MHz) 174.04 (A1), 143.05 (Q1), 142.25 (Q4), 127.25 (Q3), 117.57 (Q6), 116.69 (Q5), 115.14 (Q8), 109.22 (Q7), 90.45 (Q9), 34.36 (CQ1), 24.74 (A12), 23.58 ($-CH_2$), 21.28 ($-CH_2$), 21.14 ($-CH_2$), 20.28 ($-CH_2$), 18.13 (A13), 14.24 ($-CH_2$), 12.98 ($-CH_2$), 10.87 (CQ3), 4.96 (CQ6 and CQ8), 0.06 (A14). **ESI-IT MS (+)** ($C_{18}H_{27}ClN_3^+$, 320.19 a.m.u.) $^m/_z$: 321.12 a.m.u. (MH^+).

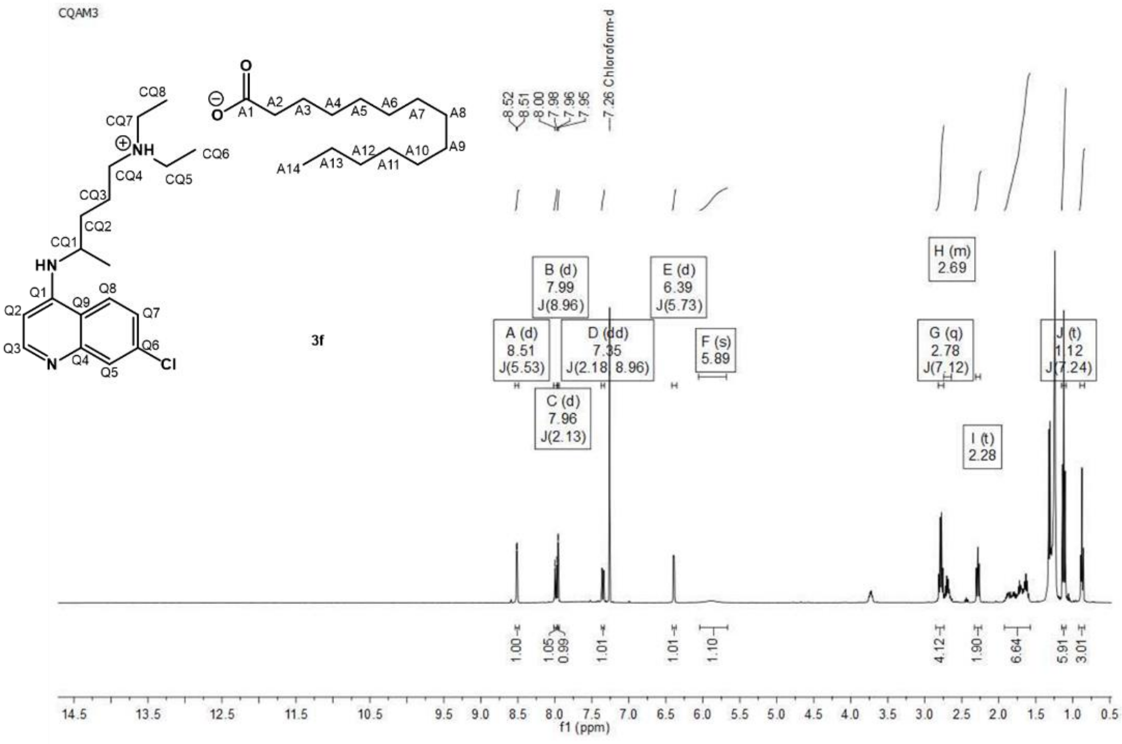

^{1}H-NMR spectrum of 3f (400 MHz, $CDCl_3$).

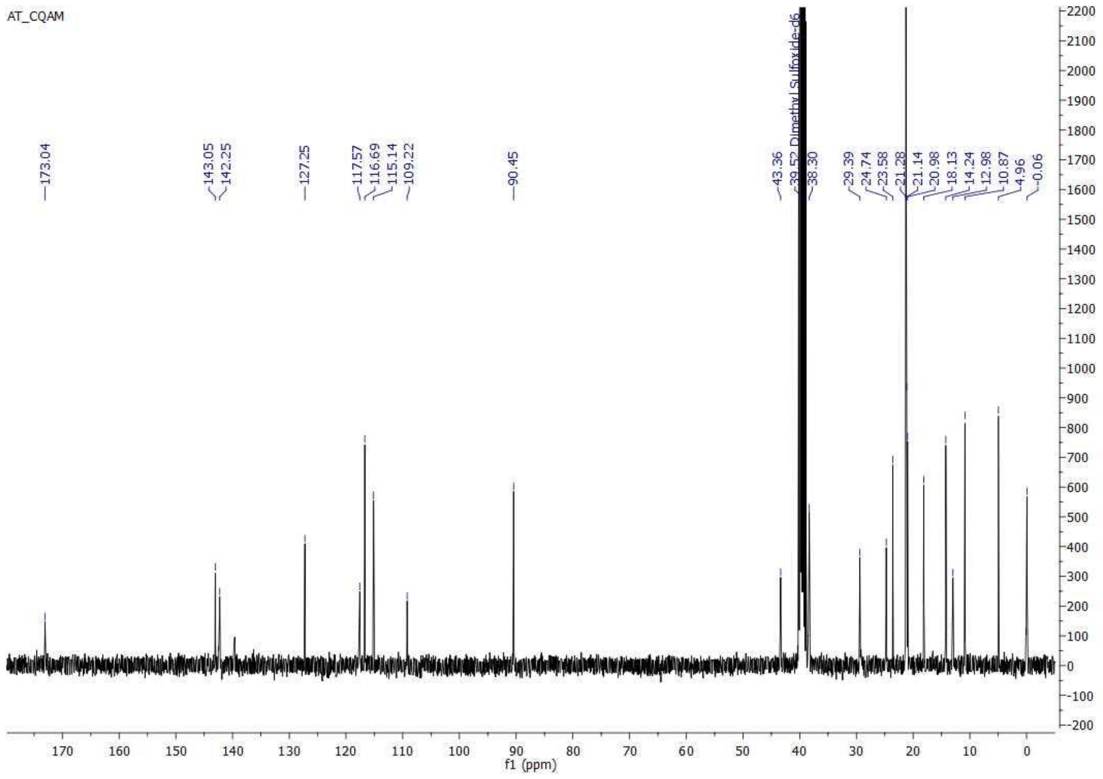

^{13}C-NMR spectrum of 3f (100 MHz, DMSO-d^{6}).

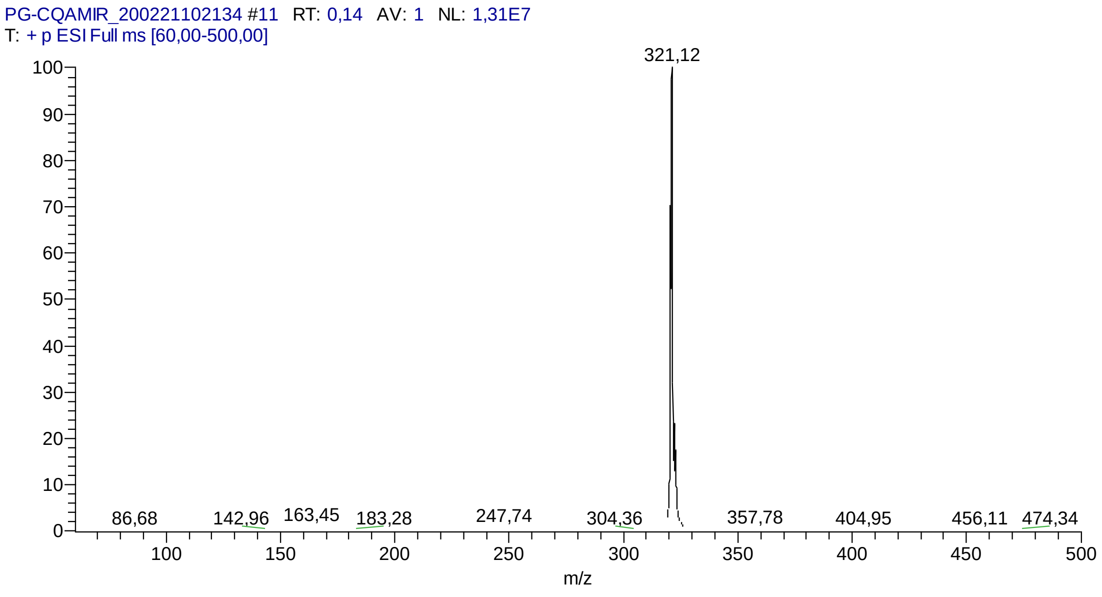

ESI-IT mass spectrum for 3f (positive mode).

3g, yellow oil; **δ**$_H$ (DMSO-d$_6$, 400 MHz) 8.35 (m, 2H, Q3 and Q8), 7.76 (d, 1H, *J*= 2.23Hz, Q5), 7.41 (dd, 1H, *J*= 9.00, 2.26 Hz, Q7), 6.91 (d, 1H, *J*= 8.01 Hz, -NH-), 6.50 (d, 1H, *J*= 5.70 Hz, Q2), 3.70 (m, 1H, CQ1), 2.42 (q, 4H, CQ5 and CQ7), 2.38 (t, 2H, *J*= 6.98Hz, CQ4), 2.16 (t, 2H, *J*= 7.36Hz, A2), 1.22 (s, 29H, -CH_2- and -CH_3-), 0.91 (t, 6H, *J*= 7.12 Hz, CQ6 and CQ8), 0.84 (t, 3H, *J*= 6.83 Hz, A16); **δ**$_C$ (DMSO-d6, 100 MHz) 174.68 (A1), 151.90 (Q1), 149.63 (Q4), 149.27 (Q3), 133.41 (Q6), 127.39 (Q5), 124.38 (Q8), 123.84 (Q7), 117.53 (Q9), 98.87 (Q2), 52.05 (CQ4), 47.64 (CQ1), 46.20 (CQ5 and CQ7), 33.87 (A2), 33.38

(CQ2), 31.33 (A10), 29.06 (-CH2-), 29.94 (-CH2-), 28.78 (-CH2-), 28.73 (-CH2-), 28.61 (-CH2-), 24.60 (-CH2-), 23.28 (-CH2-), 23.13 (CH_3), 19.88 (CQ3), 13.98 (A16), 11.52 (CQ8 and CQ6). **ESI-IT MS (+)** ($C_{18}H_{27}ClN_3^+$, 320.19 a.m.u.) $^m/_z$: 320.19 a.m.u. (MH^+).

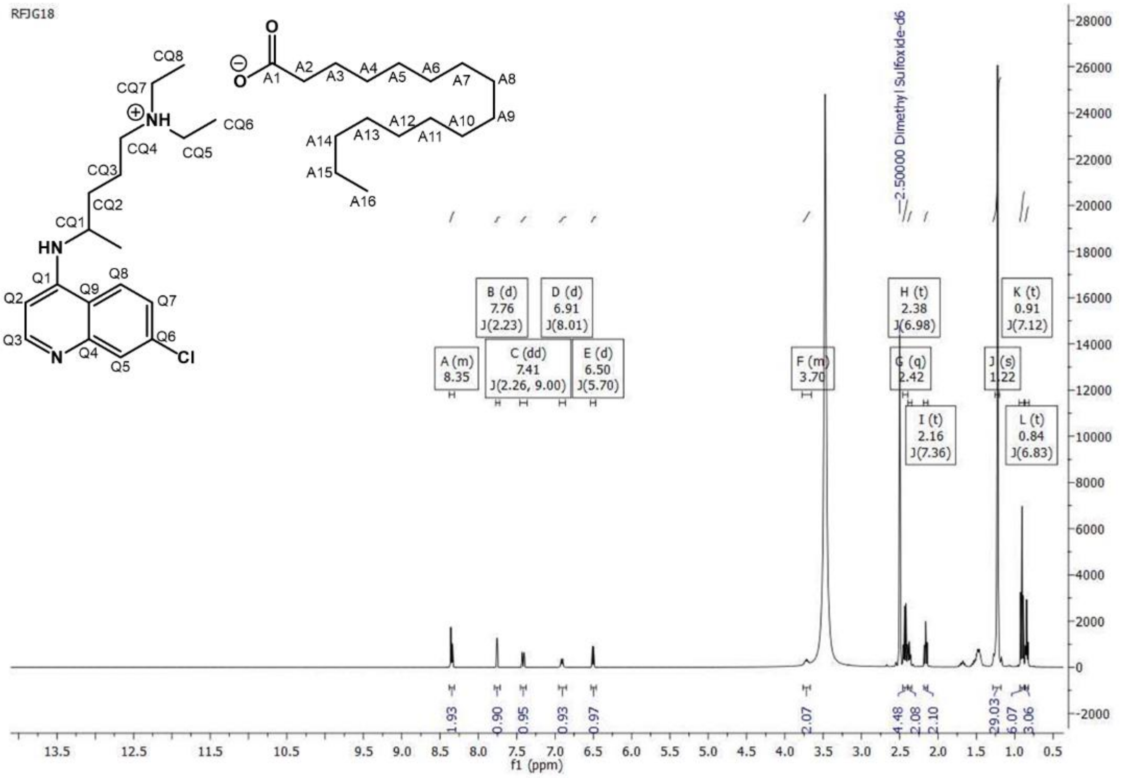

^{1}H-NMR spectrum of 3g (400 MHz, DMSO-d^6).

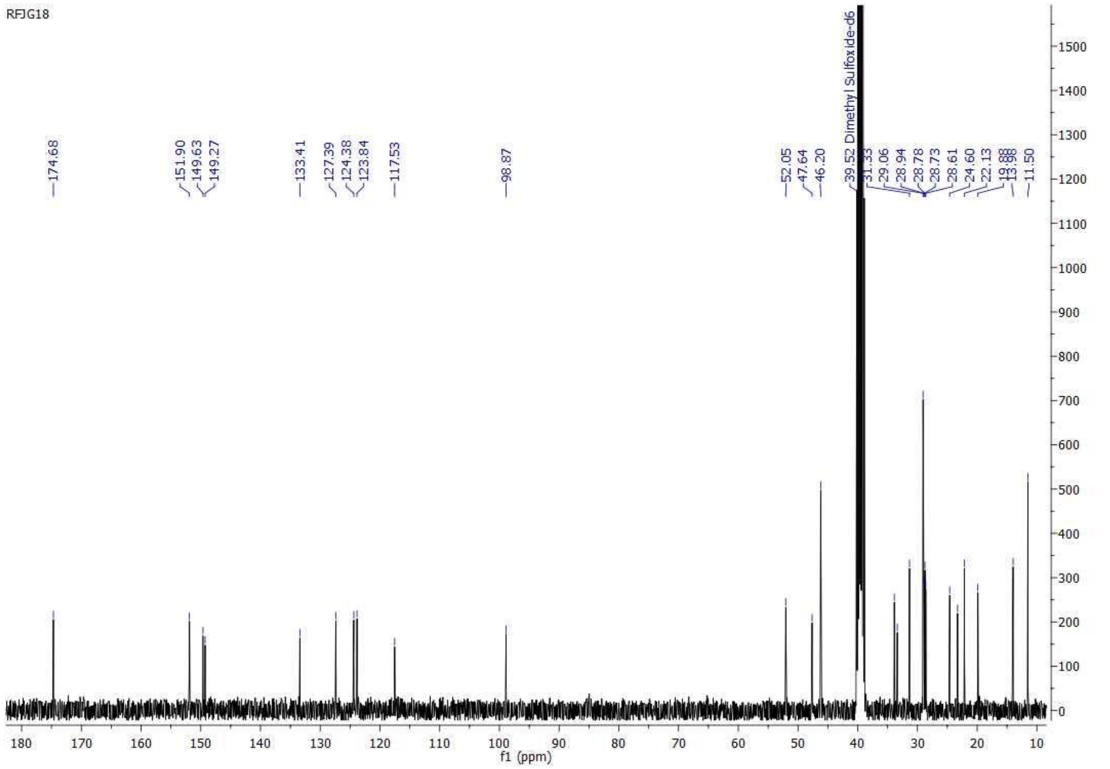

^{13}C-NMR spectrum of 3g (100 MHz, DMSO-d^6).

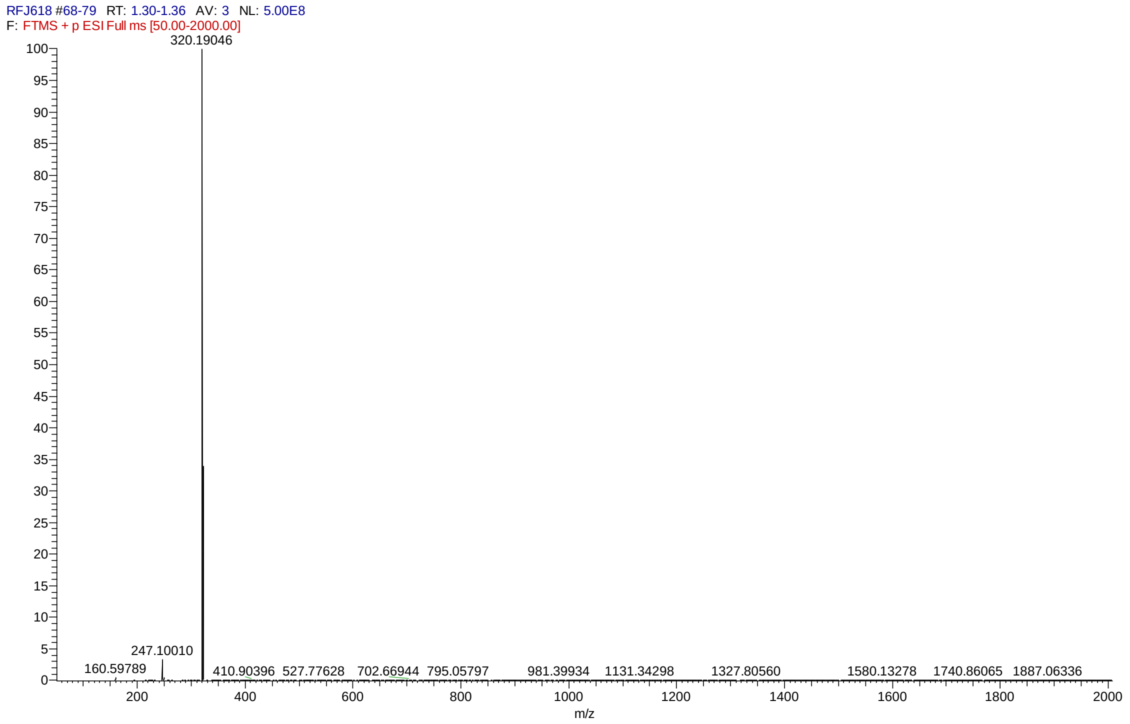

ESI-IT mass spectrum for 3g (positive mode).

1.3. Amides 4

a) Amounts of reactants used for the synthesis of 4a-e

Table S2.

**Target product**	**Solvent**	**CQ analogue (1b) / mg (mmol)**	**Fatty acid (2a-g) / mg (mmol)**	**TBTU / mg (mmol)**	**DIEA / ⚜L (mmol)**
**4a**	DCM	152 (0.61)	54 (0.61)	196 (0.61)	210 (1.20)
**4b**	DMF	60 (0.24)	36 (0.25)	87 (0.27)	95 (0.55)
**4c**	DMF	60 (0.24)	52 (0.26)	90 (0.28)	95 (0.55)
**4d**	DCM	127 (0.51)	162 (0.57)	189 (0.59)	205 (1.18)
**4e**	DMF	60 (0.24)	76 (0.27)	87 (0.27)	95 (0.55)

b) Spectral data and traces for compounds 4a-e

4a, beige solid; **m.p.** 190 °C**;** $\mathbf{R_F}$ (Ethyl acetate: Methanol 4:1) 0.67; $\boldsymbol{\delta_H}$ (DMSO-d6, 400 MHz) 8.38 (d, 1H, *J*= 5.4 Hz, Q3), 8.30 (d, 1H, *J*= 9.0 Hz, Q8), 7.78 (m, 2H, Q5 and -NH-(amide)), 7.43 (dd, 1H, *J*= 9.0, 2.3Hz, Q7), 7.38 (t, 1H, *J*= 5.3Hz, -NH-(amine)), 6.47 (d, *J*= 5.5Hz, Q2), 3.09 (m, 2H, CQ4), 2.02 (t, 2H, *J*= 7.3Hz, A2), 1.64 (m, 2H, CQ3), 1.50 (m, 4H, CQ2 e A3), 0.82 (t, 3H, *J*= 7,4Hz, A4); $\boldsymbol{\delta_C}$ (DMSO-d6, 100 MHz) 172.30 (A1), 152.20 (Q1), 150.64 (Q3), 149.41 (Q4), 133.87 (Q6), 127.79 (Q5), 124.67 (Q8), 124.46 (Q7), 117.91 (Q9), 99.11 (Q2), 42.56 (CQ1), 38.52 (CQ4), 37.86 (A2), 27.38 (CQ3), 25.66 (CQ2), 19.20 (A3), 14.09 (A4); **ESI-IT MS (+)** ($C_{17}H_{22}ClN_3O$, 319.15 a.m.u.) $^m/_z$: 320.80 a.m.u. (MH^+).

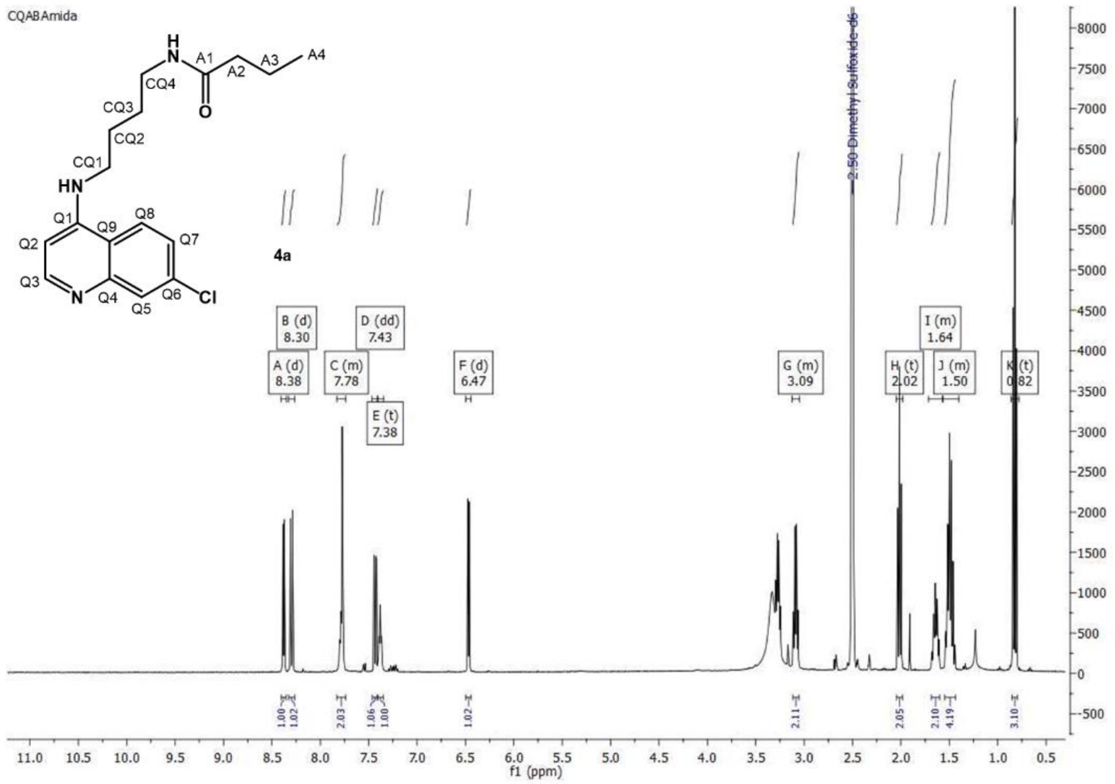

^{1}H-NMR spectrum of 4a (400 MHz, DMSO-d$_6$).

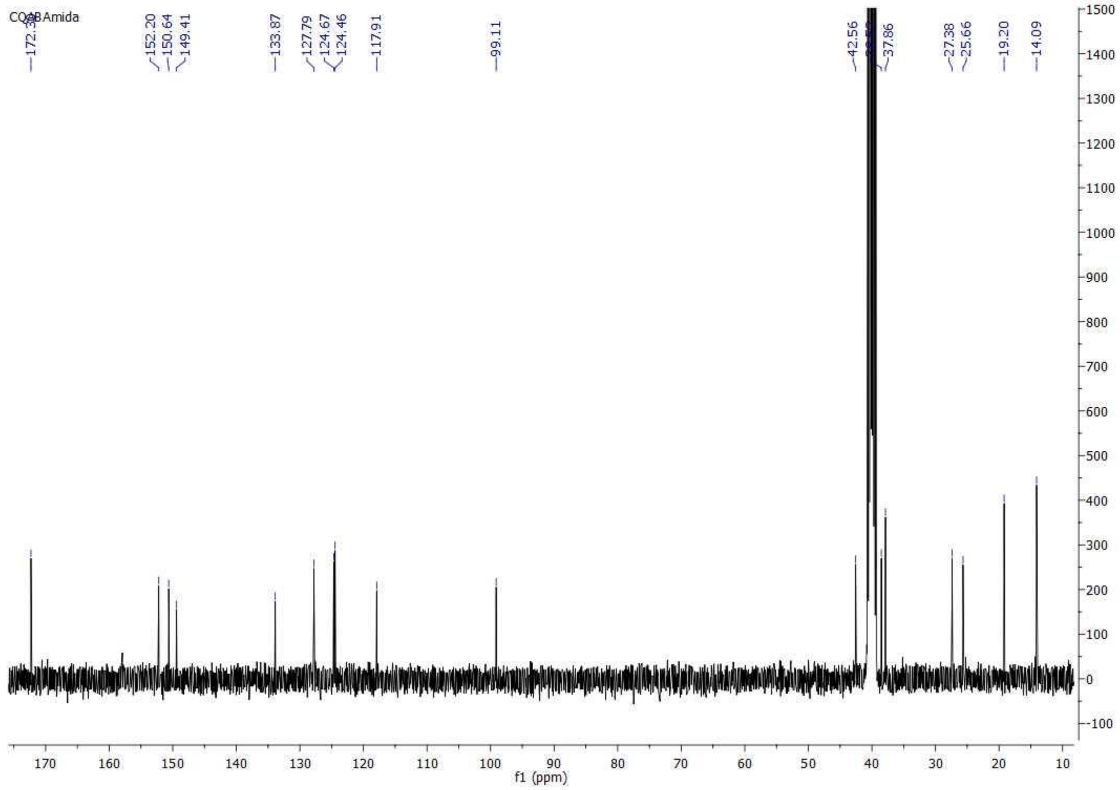

^{13}C-NMR spectrum 4a (100 MHz, DMSO-d$_6$).

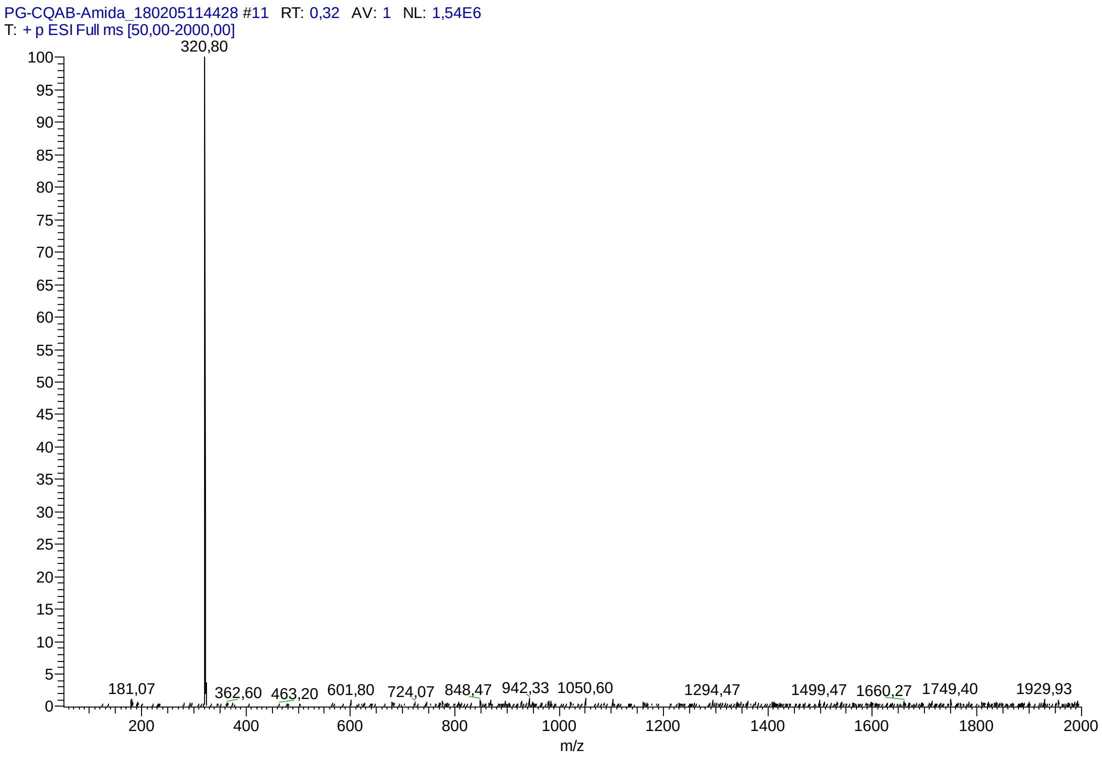

ESI-IT mass spectrum for 4a (positive mode).

4b, white solid; **m.p.** 140 °C**;** **R**$_F$ (Ethyl acetate: Methanol 4:1) 0.5; **δ**$_H$ (DMSO-d6, 400 MHz) 8.38 (d, 1H, *J*= 5.4 Hz, Q3), 8.28 (d, 1H, *J*= 9.1 Hz, Q8), 7.76 (m, 2H, Q5 and -NH-(amide)), 7.44 (dd, 1H, *J*= 9.0, 2.3Hz, Q7), 7.36 (t, 1H, *J*= 5.0Hz and -NH-(amine)), 6.47 (d, *J*= 5.5Hz, Q2), 3.09 (m, 2H, CQ4), 2.02 (t, 2H, *J*= 7,4HZ, A2), 1.64 (m, 2H, CQ3), 1.49 (m, 4H, CQ2 and A3), 1.20 (s, 8H, $-CH_2-$), 0,84 (t, 3H, *J*= 8.2Hz, A8); **δ**$_C$ ($CDCl_3$, 100 MHz) 173.95 (A1), 150.97 (Q1), 150.20 (Q3), 147.40 (Q4), 135.63 (Q6), 127.04 (Q5), 125.63 (Q8), 122.31 (Q7), 117.07 (Q9), 98.70 (Q2), 43.32 (CQ1), 38.84 (CQ4), 36.83 (A2), 31.69 (A6), 29.28 (A5), 29.02 (A4), 28.19 (CQ3), 25.83 (CQ2), 25.09 (A3), 22.60 (A7), 14.12 (A8); **ESI-IT MS (+)** ($C_{21}H_{30}ClN_3O$, 375.21 a.m.u.): $^m/_z$ 376.73 a.m.u. (MH^+).

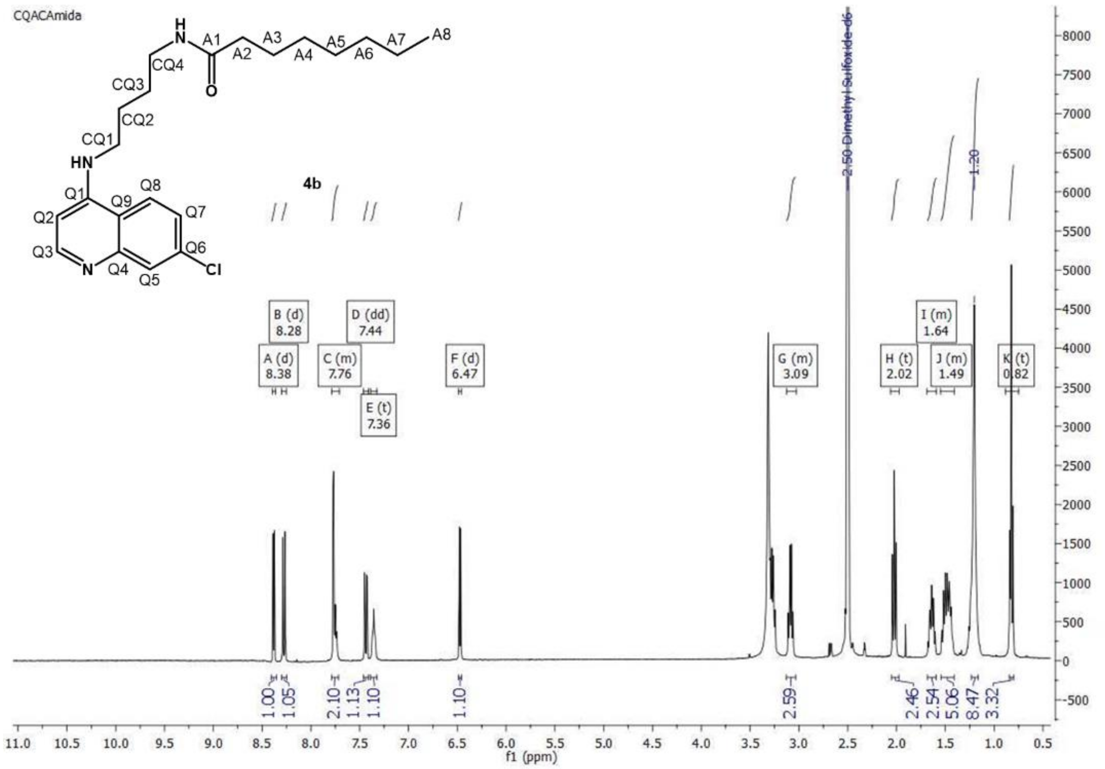

^{1}H-NMR spectrum of 4b (400 MHz, DMSO-d_6).

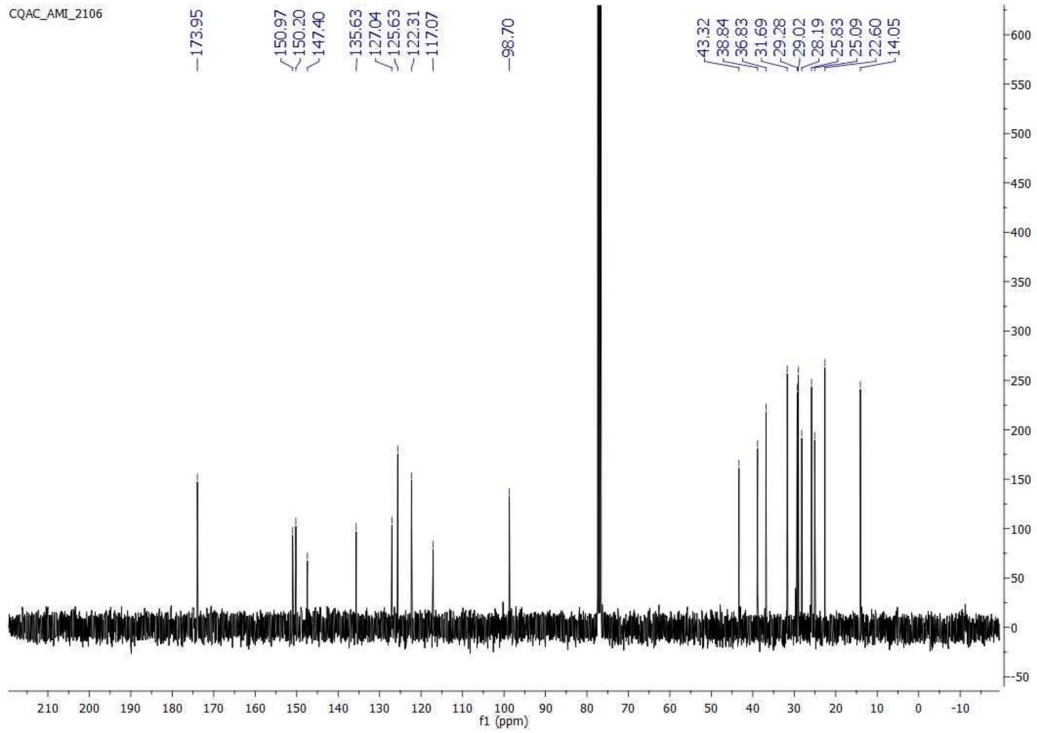

^{13}C-NMR spectrum of 4b (100 MHz, $CDCl_3$).

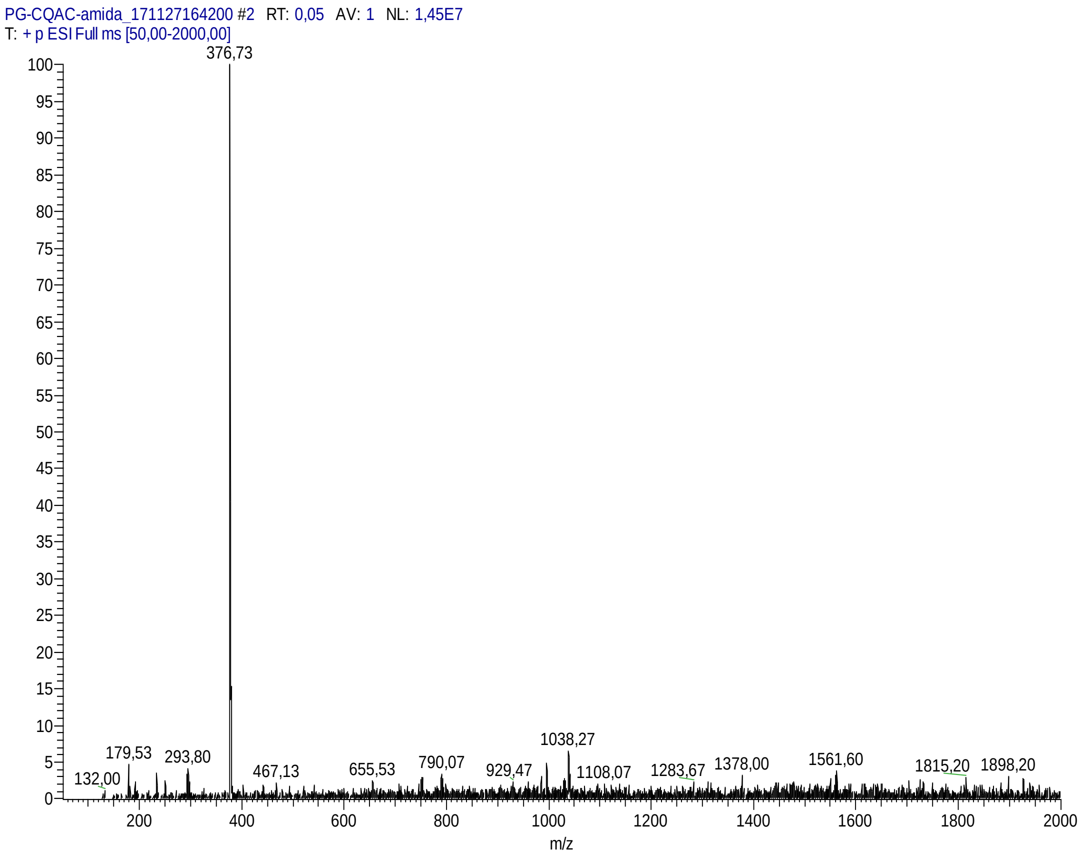

ESI-IT mass spectrum for 4b (positive mode).

4c, white solid; **m.p.** 140 °C**; R$_F$** (Ethyl acetate: Methanol 4:1) 0.64; **δ$_H$** (DMSO-d6, 400 MHz) 8.37 (d, 1H, *J*= 5.4 Hz, Q3), 8.28 (d, 1H, *J*= 9.1 Hz, Q8), 7.77 (m, 2H, Q5 and -NH- (amide)), 7.45 (dd, 1H, *J*= 9.0, 2.3Hz , Q7), 7.32 (t, 1H, *J*= 5.2Hz, -NH-(amine)), 6.46 (d, *J*= 5.5Hz, Q2), 3.26 (m, 1H, CQ1), 3.09 (m, 2H, CQ4), 2.02 (t, 2H, *J*= 7.4Hz, A2), 1.64 (m, 2H,CQ2), 1.48 (m, 4H, CQ3 and A3), 1,22 (s, 16H, -CH_2-), 0,84 (t, 3H, *J*= 6.9Hz, A12); **δ$_C$** ($CDCl_3$, 100 MHz) 174.24 (A1), 126.34 (Q5), 43.74 (CQ1), 38.75 (CQ4), 36.79 (A2), 31.90 (A10), 29.62 (A8), 29.61 (A7), 29.52 (A6), 29.39 (A9), 29.33 (A4), 28.28 (A5), 25.85 (CQ2), 24.68 (A11), 14.11 (A12); **ESI-IT MS (+)** ($C_{25}H_{38}ClN_3O$, 431.27 a.m.u.): $^m/_z$ 432.67 a.m.u. (MH^+).

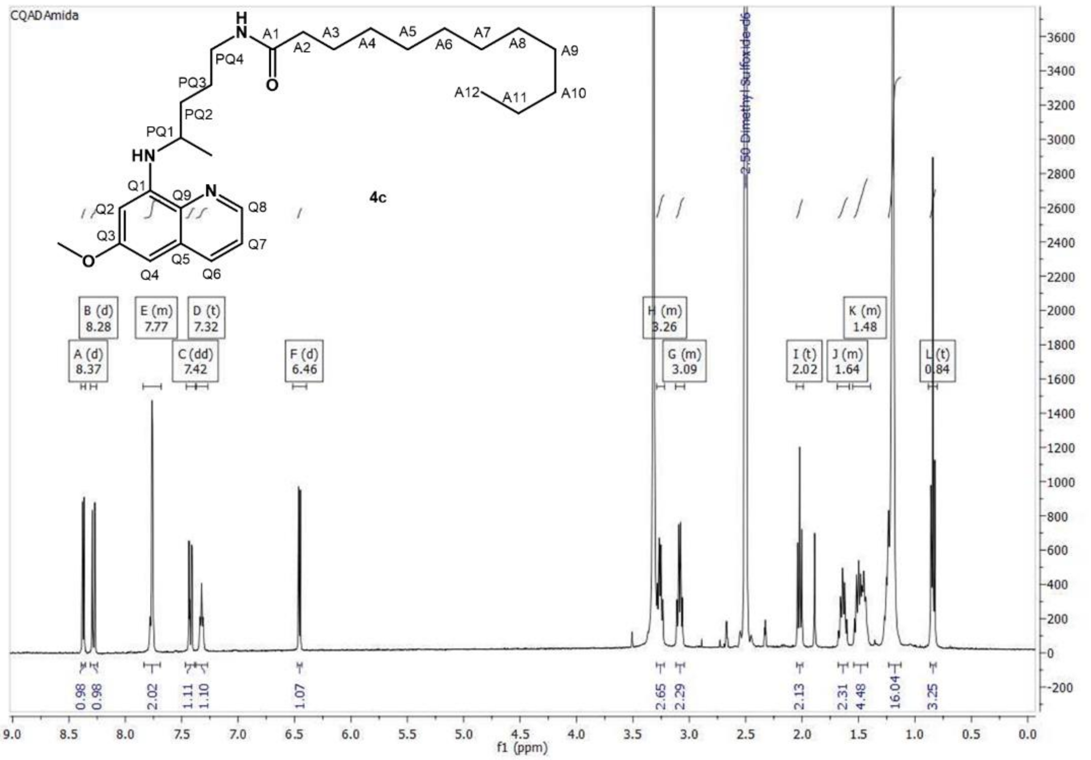

^{1}H-NMR spectrum of 4c (400 MHz, DMSO-d_6).

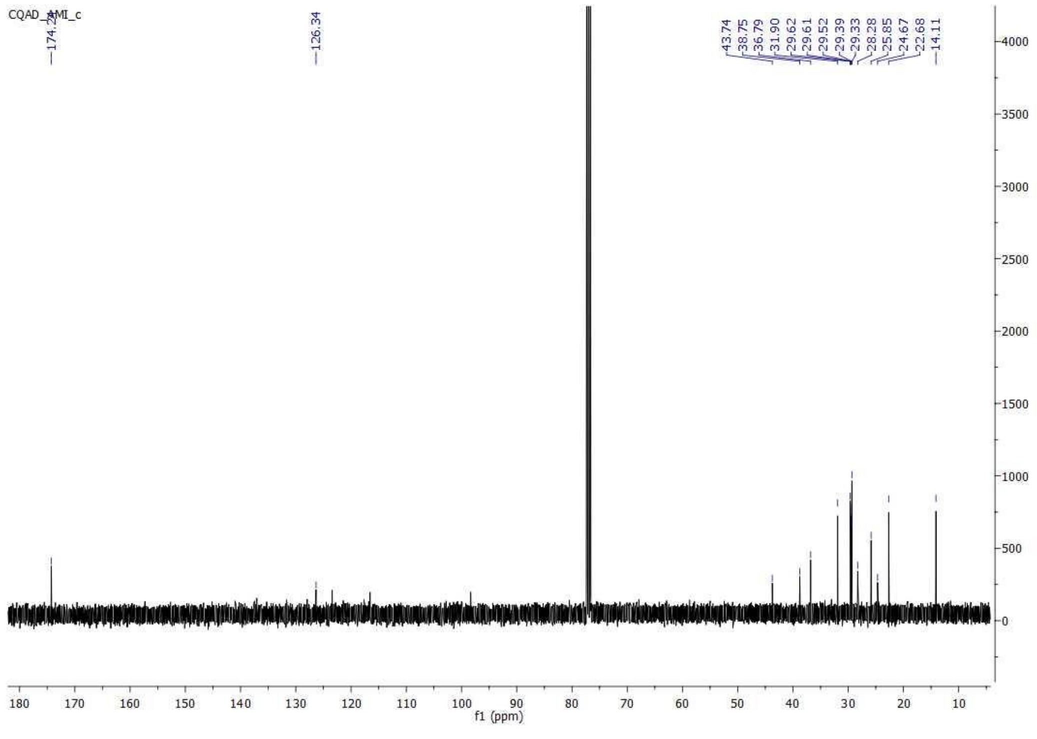

^{13}C-NMR spectrum of 4c (100 MHz, $CDCl_3$).

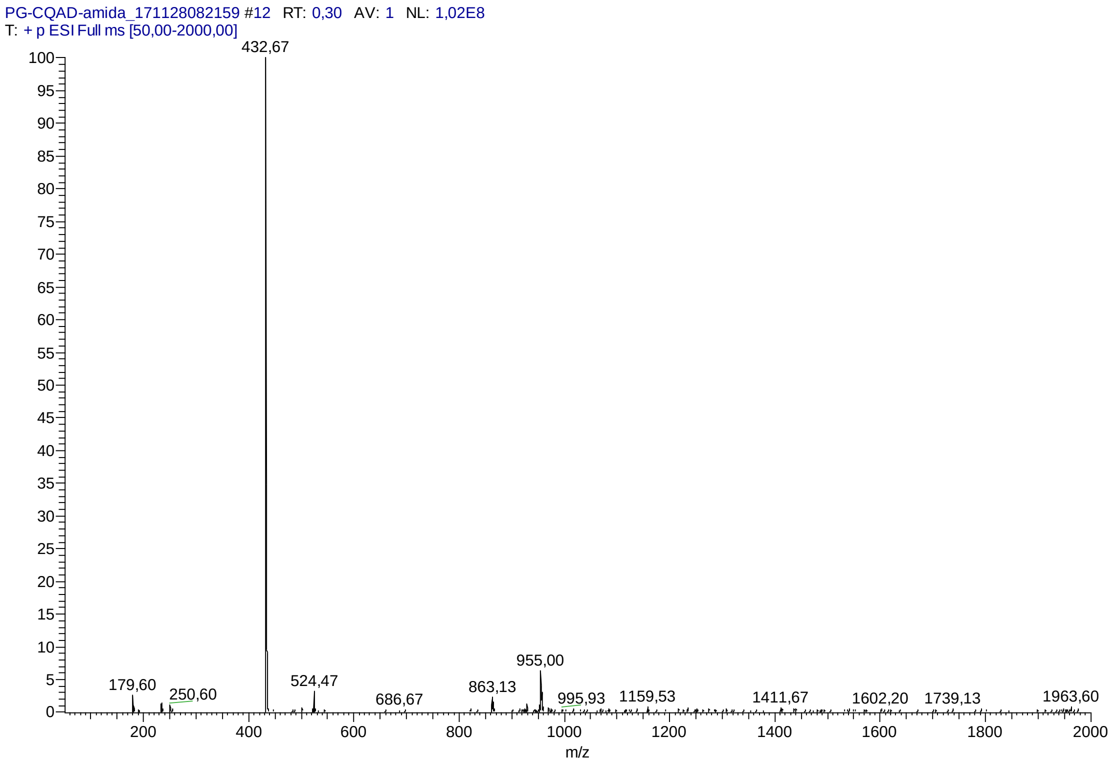

ESI-IT mass spectrum for **4c** (positive mode).

4d, white solid; **m.p.** 110 °C**;** $\mathbf{R_F}$ (Ethyl acetate: Methanol 4:1) 0.53; $\boldsymbol{\delta_H}$ (DMSO-d6, 400 MHz) 8.37 (d, 1H, *J*= 5.4 Hz, Q3), 8.26 (d, 1H, *J*= 9.0 Hz, Q8), 7.76 (m, 2H, Q5 and -NH- (amide)), 742 (dd, 1H, *J*= 9.0, 2.1Hz, Q7), 7.30 (t, 1H, *J*= 5.4Hz, -NH-(amine)), 6.46 (d, 1H, *J*= 5.5Hz, Q2), 3.09 (m, 2H, CQ4), 2.02 (t, 2H, *J*= 7.4Hz, A2), 1.64 (m, 2H, CQ2), 1.49 (m, 4H, CQ3 and A3), 1.22 (d, 28H, -CH_2-), 0.84 (t, 3H, *J*= 74Hz, A18); $\boldsymbol{\delta_C}$ ($CDCl_3$, 100 MHz) 174.03 (A1), 151.48 (Q1), 136.08 (Q6), 125.85 (Q8), 122.59 (Q7), 116.85 (Q9), 98.65 (Q2), 43.44 (CQ1), 38.81 (CQ4), 36.82 (A2), 31.92 (A16), 29.70 (A6-14), 29.52 (A15), 29.38 (A5), 28.22 (A4), 27.17 (CQ3), 25.84 (CQ2), 24.55 (A3), 22.69 (A17), 14.12 (A18); **ESI-IT MS (+)** ($C_{31}H_{50}ClN_3O$, 515.36 g/mol): $^m/_z$ 516.55 a.m.u. (MH^+).

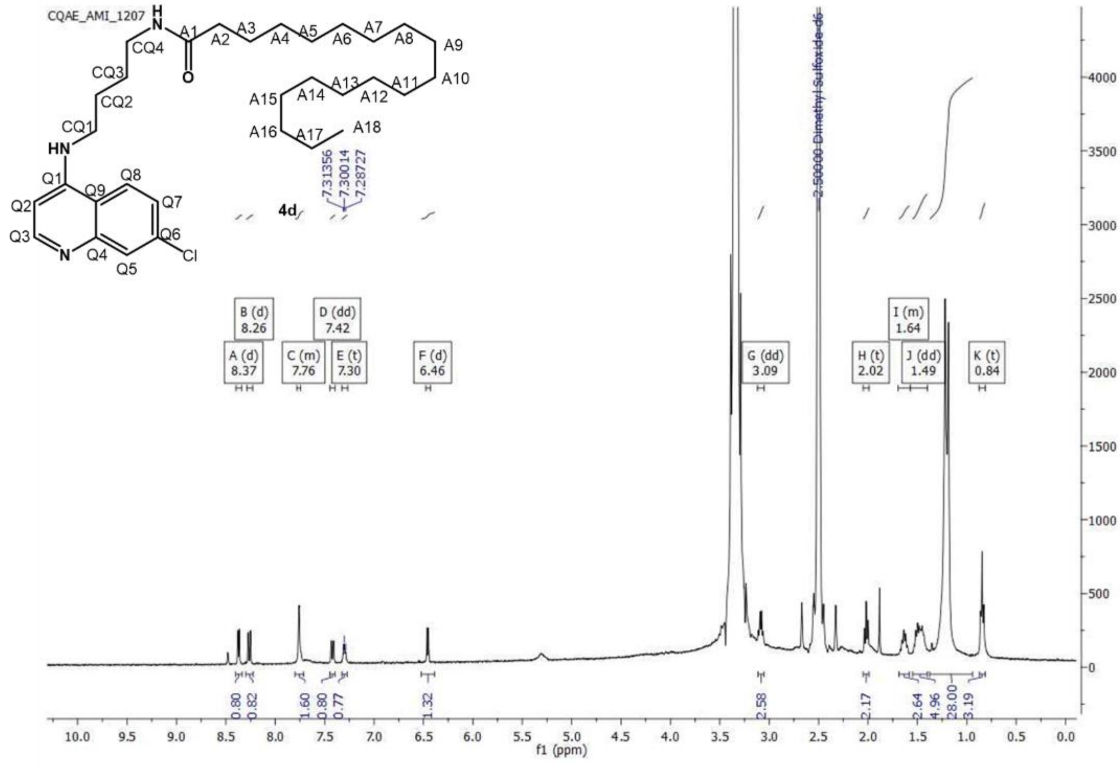

^{1}H-NMR spectrum of 4d (400 MHz, DMSO-d$_6$).

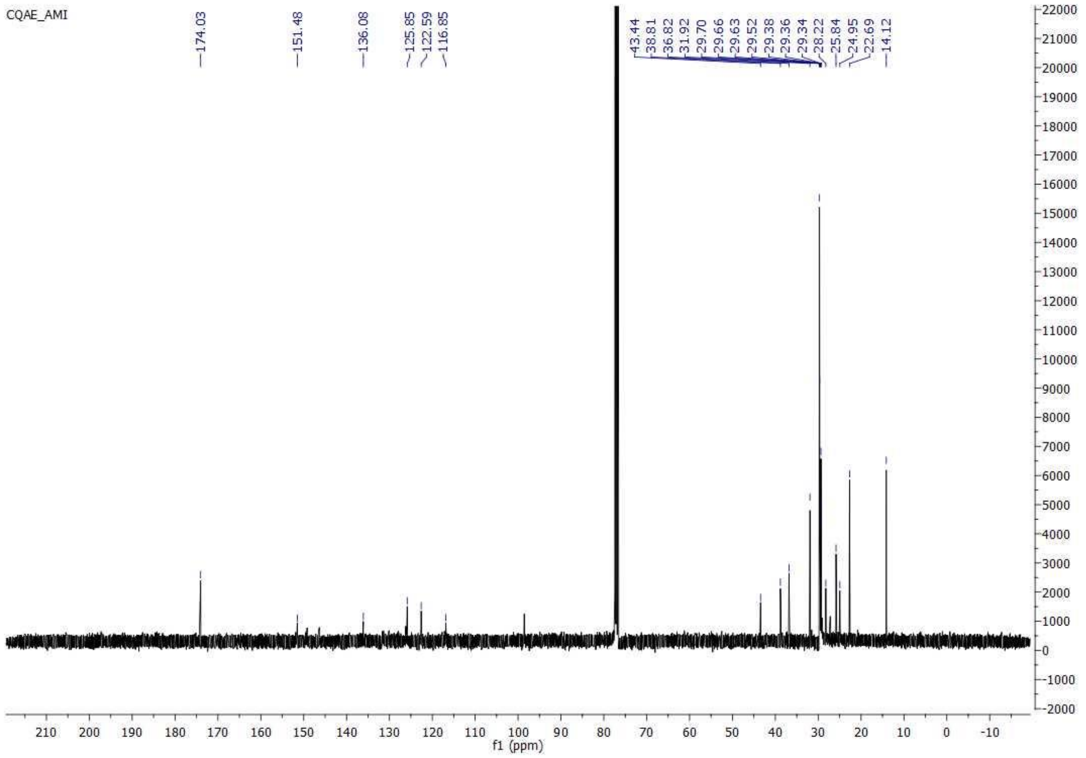

^{13}C-NMR spectrum of 4d (100 MHz, CDCl$_3$).

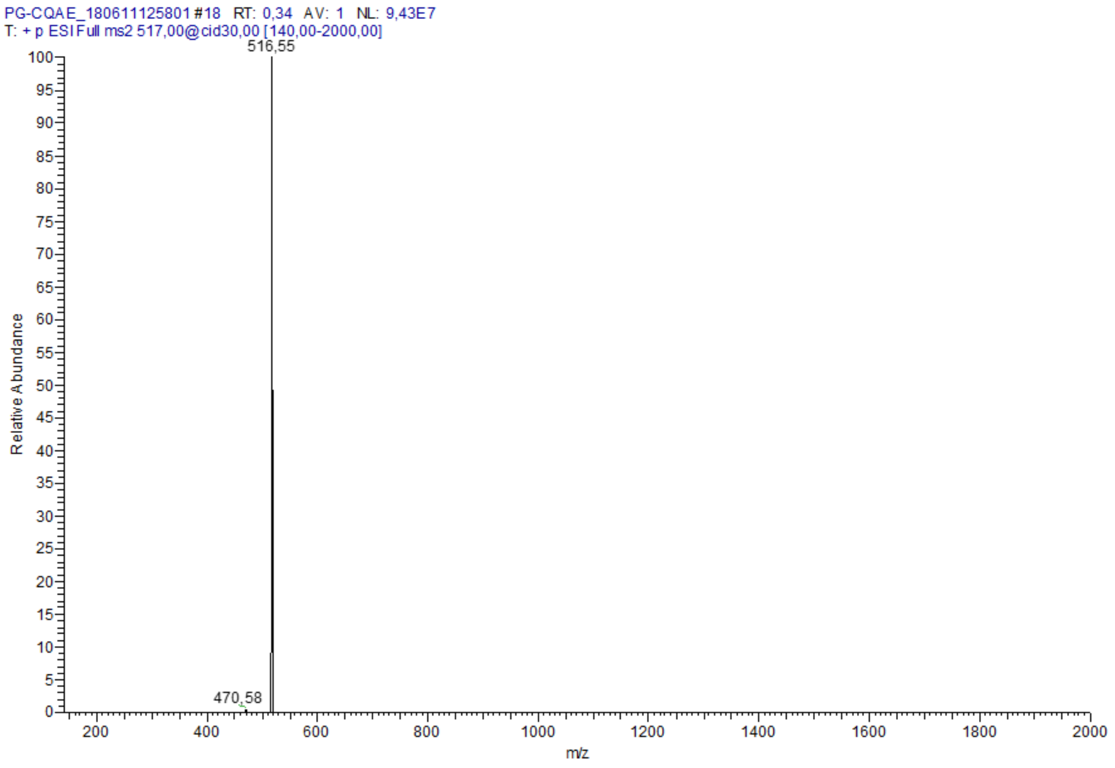

ESI-IT mass spectrum for 4d (positive mode).

4e, white solid; **m.p.** 140 °C**;** $\mathbf{R_F}$ (Ethyl acetate: Methanol 4:1) 0.69; $\boldsymbol{\delta_H}$ (DMSO-d6, 400 MHz) 8.38 (d, 1H, *J* = 5.5Hz, Q3), 8.29 (d, 1H, *J*= 9,1 Hz, Q8), 7.77 (d, 1H, *J*= 2,2Hz, Q5), 7.75(t, 1H, *J*= 5.6 Hz,-NH-(amide)), 7.45 (m, 2H, Q7 and -NH-(amine)), 6.49 (d, *J*=5.6Hz, Q2), 5.30 (m, 2H, Ha e Hb), 3.09 (m, 2H, CQ1), 1.98 (m, 6H, A2, A8 and A11), 1.64 (m, 2H, CQ2), 1.48 (m, 4H, CQ3 and A3), 1.22 (s, 20H, -CH_2-), 0.84 (t, 3H, *J*=8Hz, A18); $\boldsymbol{\delta_C}$ ($CDCl_3$, 100 MHz) 173.95(A1), 151.17(Q1), 149.83 (Q3), 147.03 (Q4), 135.79 (Q6), 130.03 (A9), 129.71 (A10), 126.71 (Q5), 125.70 (Q8), 122.48 (Q7), 117.00 (Q9), 98.65 (Q2), 43.37 (CQ1), 38.84 (CQ4), 36.81 (A2), 31.93 (A11), 31.90 (A8), 29.70 (A7 and A12), 29.52 (A16), 29.31 (A6, A14 and A13), 29.16 (A15), 28.19 (A5), 27.23 (A4), 27.17 (CQ3), 25.83 (CQ2), 25.05 (A3), 22.68 (A17), 14.12 (A18); **ESI-IT MS (+)** ($C_{31}H_{48}ClN_3O_3$, 513.35 g/mol): $^m/_z$ 514.62 a.m.u. (MH^+).

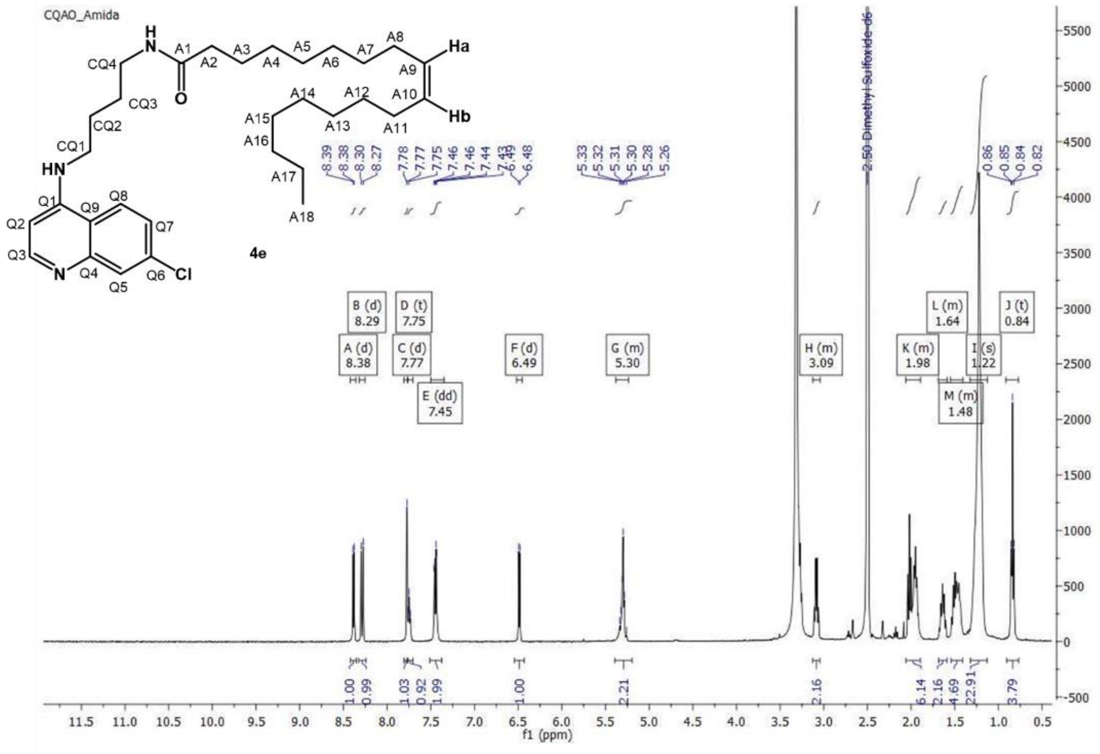

^{1}H-NMR spectrum of 4e (400 MHz, DMSO-d$_6$).

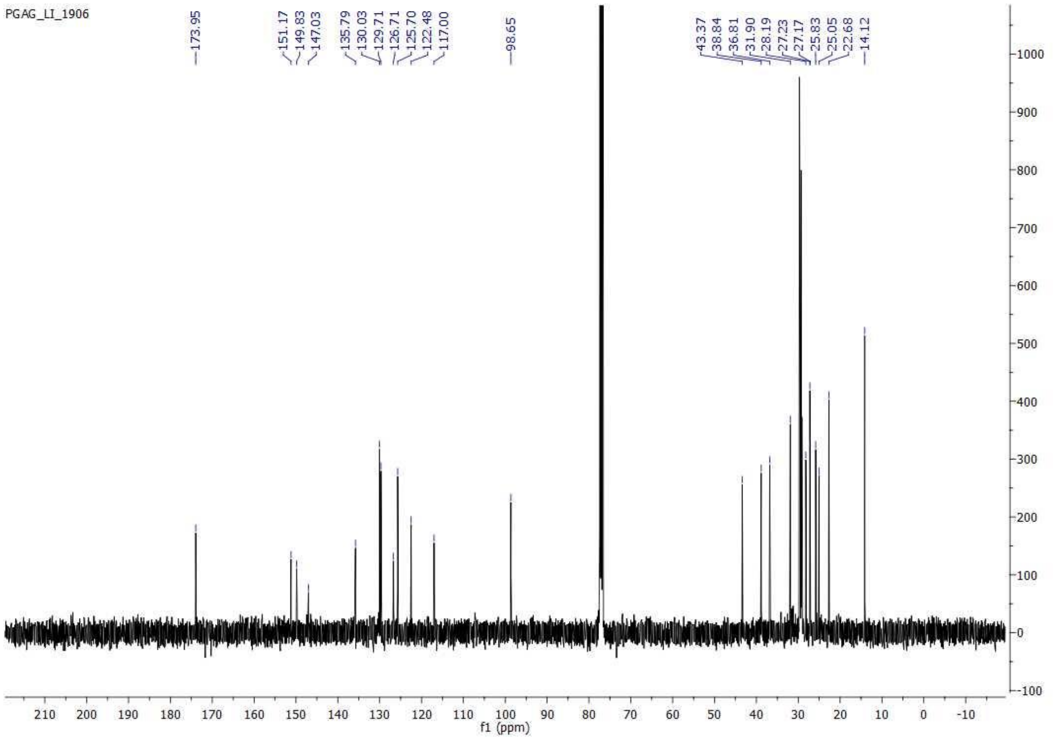

^{13}C-NMR spectrum of 4e (100 MHz, CDCl$_3$).

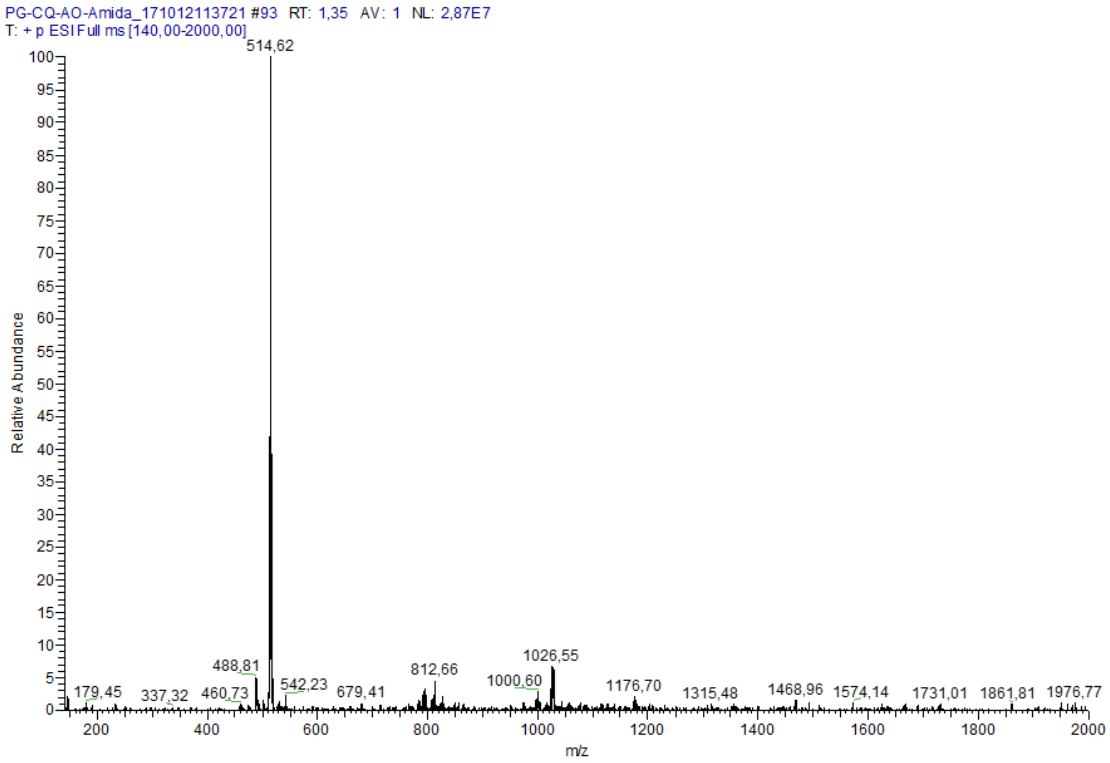

ESI-IT mass spectrum for 4e (positive mode).

1.4. Figure S1

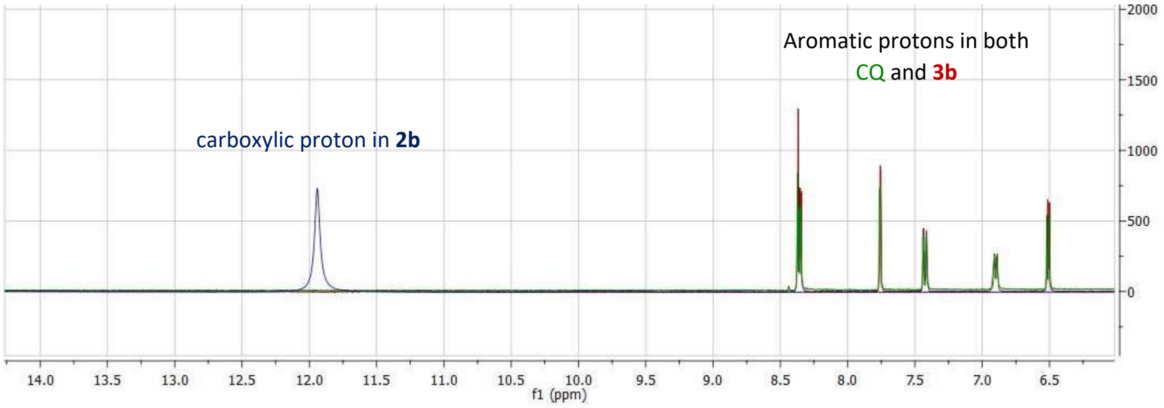

Figure S1. Superimposed ^{1}H NMR spectra of octanoic acid **2b** (blue), basic chloroquine **1a** (green), and their derived ionic liquid **3b** (red); the carboxylic proton peak at ca. 12 ppm is exclusively observed in the spectrum of **2b**, while missing in the spectrum of **3b**, thus confirming the complete transfer of the acidic proton to the basic antimalarial drug.

2. Simultaneous thermogravimetric analysis

2.1. Thermograms for ionic liquids 3

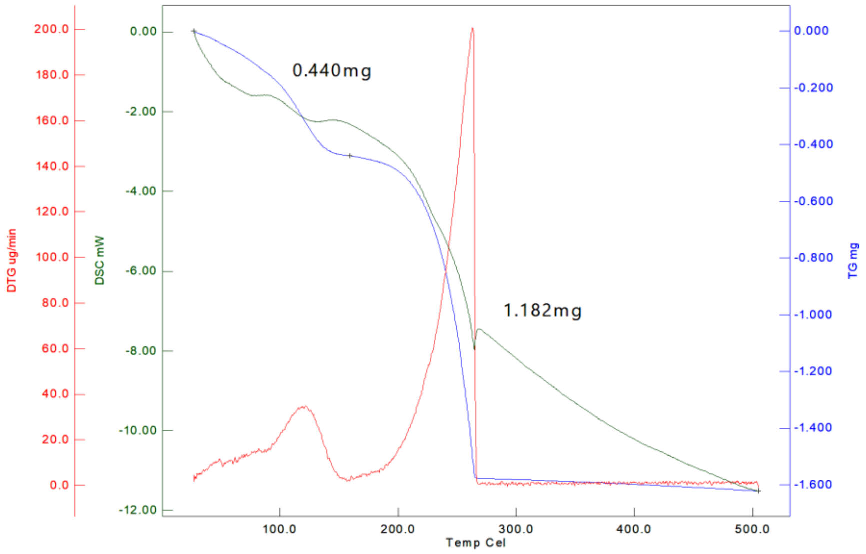

Thermogram of **3a**.

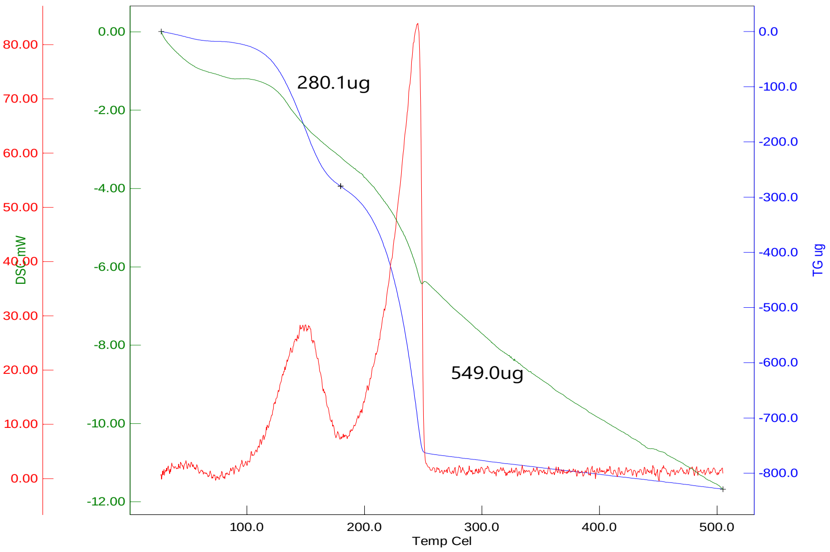

Thermogram of **3b**.

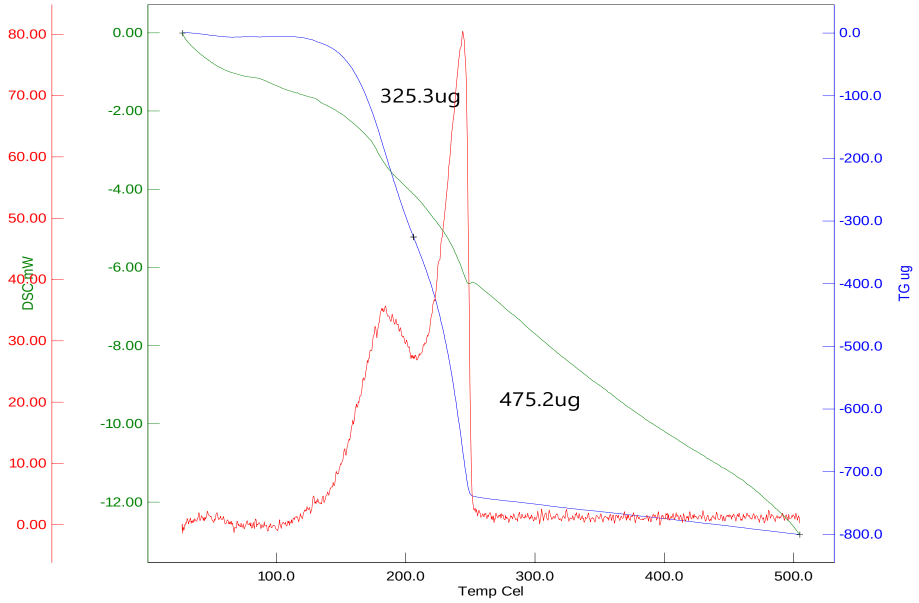

Thermogram of **3c**.

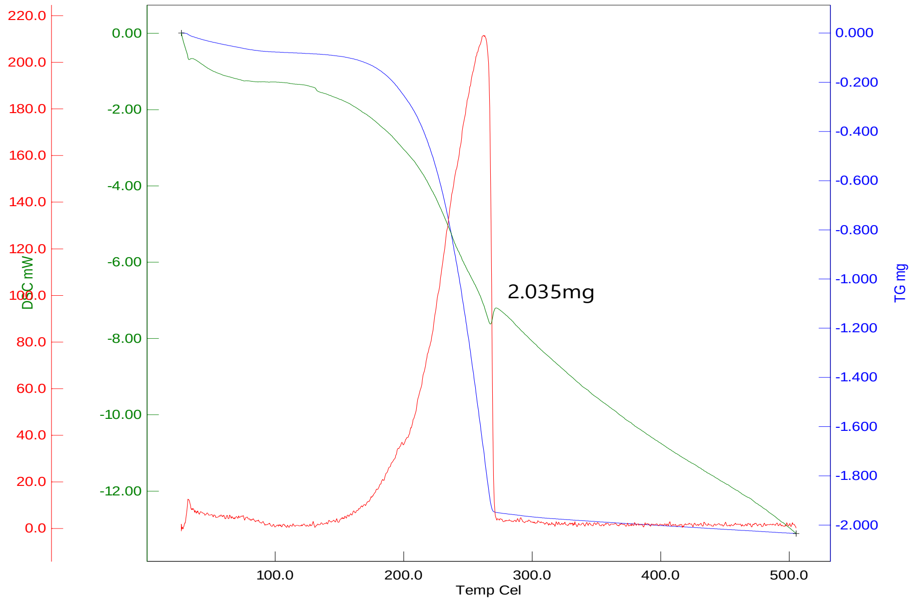

Thermogram of **3d**.

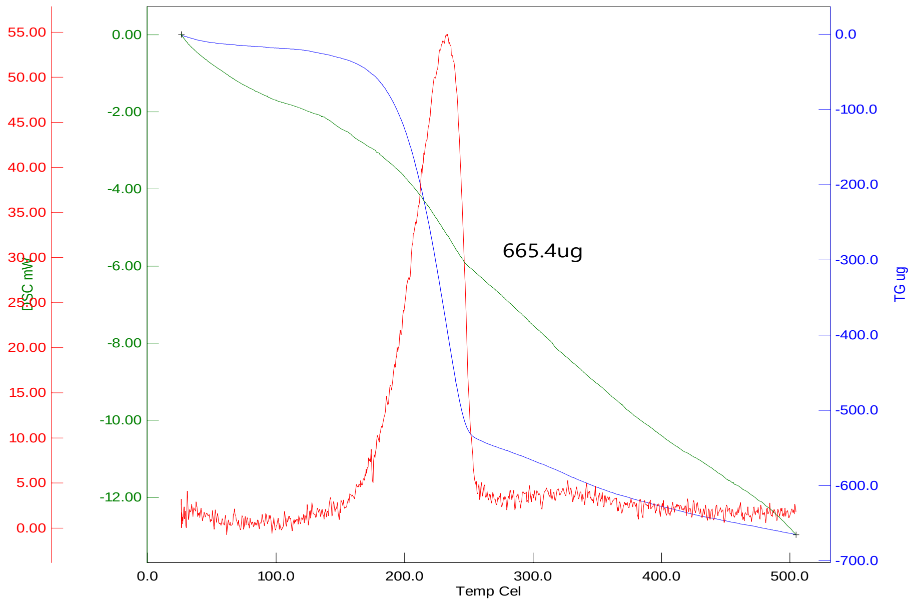

Thermogram of **3e**.

2.2. Thermograms for covalent conjugates 4

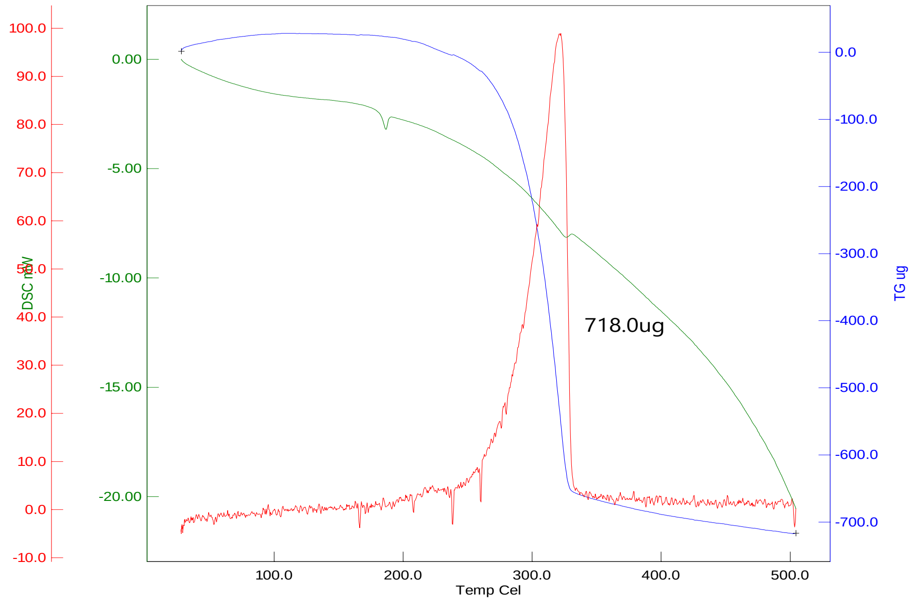

Thermogram of **4a**.

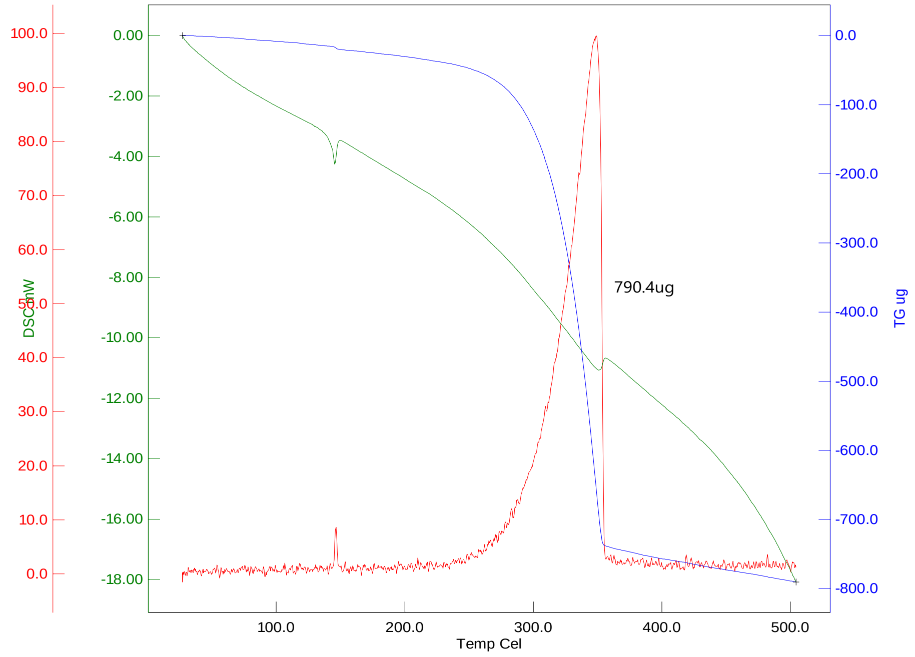

Thermogram of **4b**.

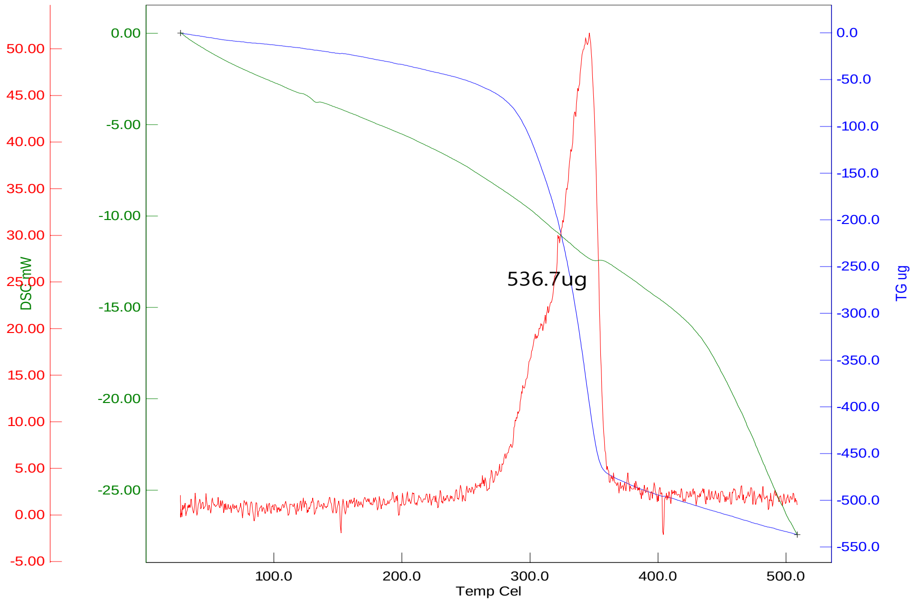

Thermogram of **4c**.

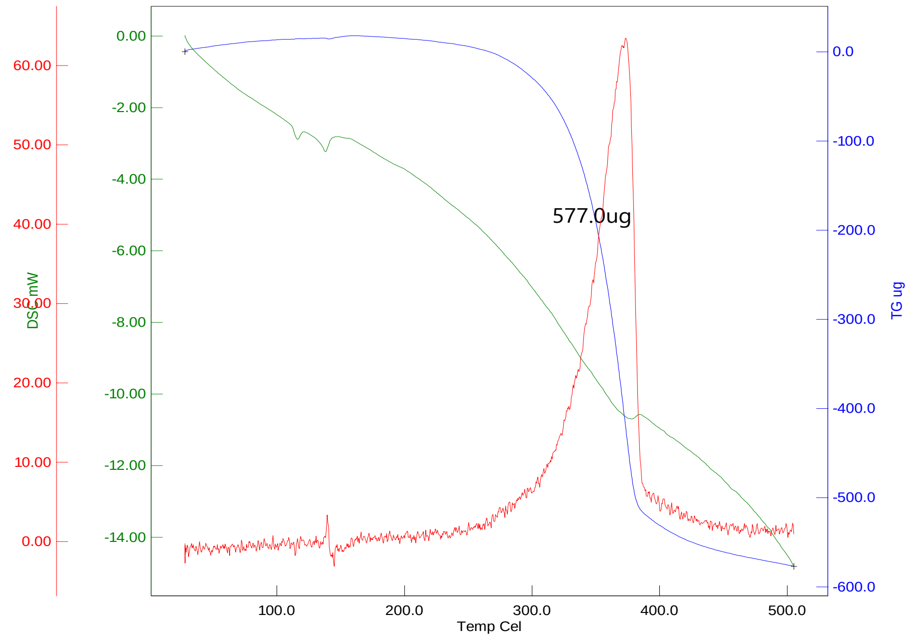

Thermogram of **4d**.

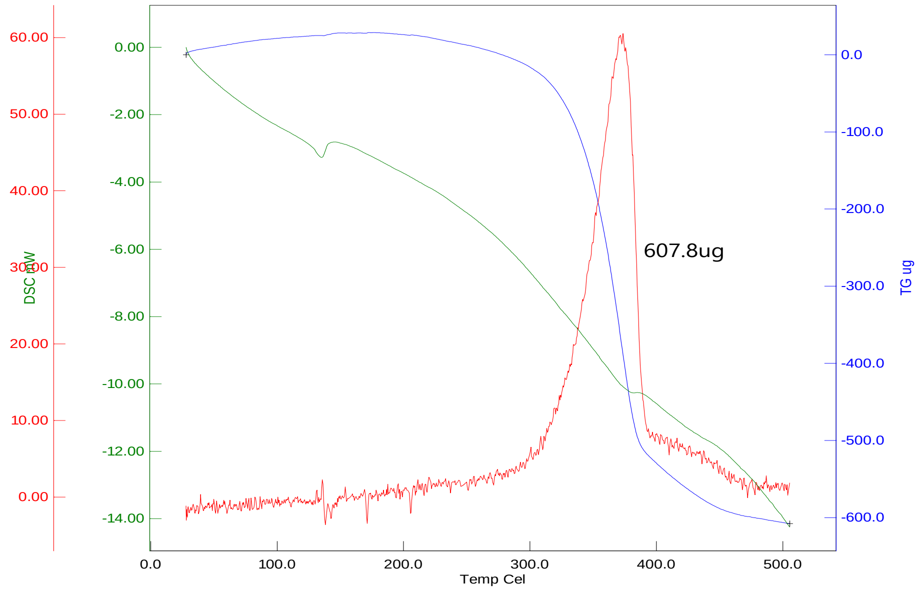

Thermogram of **4e**.

3. Surface tension measurements

The surface tension of a saturated solution of SAIL **3c** in water, shown in Figure S2, was evaluated yielding a value of 29.8 ± 0.3 mN·m^{-1} (two independent solutions; three measurements per solution).

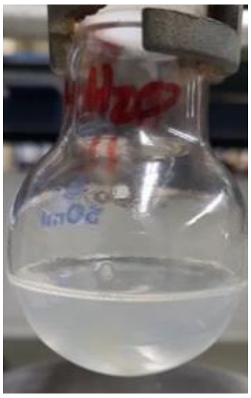

Figure S2. Appearance of a saturated solution of **3c** in water, displaying turbidity and foam formation.

Figure S3 and Table S1 show, respectively, the surface tension curves and the obtained *cmc* values for CTAB/**3c** solutions with increasing molar fraction of **3c**.

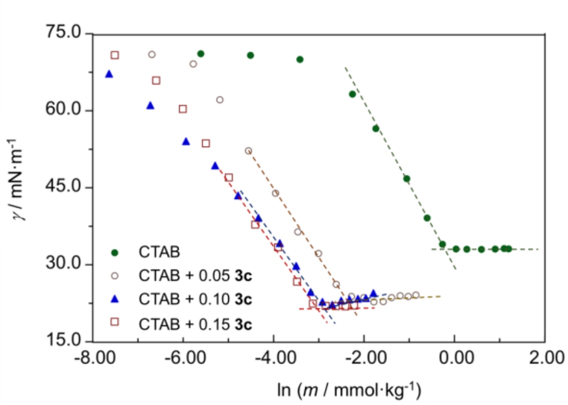

Figure S3. Surface tension plots and *cmc* determination, at 25.0 °C, of aqueous CTAB/SAIL **3c** mixtures for increasing molar fraction of **3c** (0.05, 0.10. and 0.15).

Table S1. Values for *cmc* and surface tension at the *cmc* (γ_{cmc}) for CTAB/SAIL **3c** with increasing molar fraction of **3c.**

**molar fraction of 3c**	***cmc* / mmol·kg^{-1}**	**γ_{cmc} / mN·m^{-1}**
0	0.84	33.0
0.05	0.11	23.6
0.10	0.057	22.0
0.15	0.047	21.9
